# Plant‑mediated silver quantum dots from *Drimia maritima*: antioxidant modulation and ROS‑induced apoptosis in breast cancer cells

**DOI:** 10.1038/s41598-026-53467-7

**Published:** 2026-05-21

**Authors:** Narges Jalali, Fateme Aghamir, Ghasem Eghlima, Zinab Moradi Alvand

**Affiliations:** 1 Department of Agriculture, Medicinal Plants and Drugs Research Institute, Shahid Beheshti University, Tehran, Iran; 2 Department of Phytochemistry, Medicinal Plants and Drugs Research Institute, Shahid Beheshti University, Tehran, Iran

**Keywords:** Microwave-assisted extraction, Quantum dots, *Drimia maritima* (L.) Stearn, MCF-7 cells, Biochemistry, Biotechnology, Cancer, Nanoscience and technology, Plant sciences

## Abstract

Green nanotechnology offers a sustainable approach to improving the therapeutic efficacy of plant‑derived bioactive compounds while reducing environmental and biological toxicity. In this study, silver quantum dots (Ag QDs) were synthesized via an eco‑friendly green route using the aqueous bulb extract of *Drimia maritima* (L.) Stearn (*D. maritima)*, which served as a natural reducing and stabilizing agent. Physicochemical characterization confirmed the successful formation of well‑defined nanoscale Ag QDs with appropriate structural features. The antioxidant potential of the biosynthesized Ag QDs was systematically evaluated using various in vitro assays, including DPPH, ABTS, nitric oxide scavenging, ferric reducing antioxidant power (FRAP), and metal‑chelating activity. The results revealed a dual antioxidant behavior: Ag QDs showed enhanced free‑radical scavenging capacity, whereas the crude extract exhibited stronger reducing and chelating activities. The anticancer efficacy of Ag QDs was assessed in MCF‑7 human breast cancer cells. MTT assay results revealed a significant cytotoxic effect, with an IC₅₀ value of approximately 14.7 µg/mL, approaching that of cisplatin and markedly lower than that of the crude extract. Mechanistic studies indicated that Ag QDs induced excessive intracellular ROS generation, leading to mitochondrial dysfunction and activation of the intrinsic apoptotic pathway, characterized by pronounced caspase‑9 and caspase‑3 activation. These effects were accompanied by modulation of apoptosis‑ and redox‑related gene expression, favoring pro‑apoptotic signaling. Confocal microscopy further confirmed apoptotic morphology, including chromatin condensation and cytoskeletal disruption. Overall, *D. maritima*‑derived Ag QDs emerge as potent pro‑oxidant nanostructures capable of inducing ROS‑mediated mitochondrial apoptosis, highlighting their promise as a sustainable nanotherapeutic platform for breast cancer treatment.

## Introduction

Medicinal plants continue to play a pivotal role in modern drug discovery due to their rich reservoir of bioactive secondary metabolites with therapeutic potential. Among them, *Drimia maritima* (L.) Stearn (commonly known as squill) is a perennial geophyte native to the Mediterranean region and traditionally valued for its cardiotonic, anti‑inflammatory, and anticancer effects^1^. The bulbs of *D. maritima* contain high levels of pharmacologically active constituents such as bufadienolides, flavonoids, and phenolic compounds, all of which have demonstrated notable bioactivity in various disease models^2^.

Despite its promising pharmacological profile, the clinical translation of plant‑derived bioactives remains limited, primarily due to their suboptimal physicochemical properties and safety considerations, emphasizing the need for advanced delivery platforms^3^. Natural products and their derivatives currently account for nearly 60% of anticancer agents in clinical use or under investigation, highlighting the central contribution of plant‑derived metabolites to oncology^4^. These metabolites exert anticancer effects by modulating key cellular processes, including proliferation, apoptosis, and oxidative stress. Owing to their structural diversity and multifaceted mechanisms of action, plant‑based compounds particularly those from *D. maritima* have gained considerable attention as promising candidates for anticancer drug development. Consequently, ongoing research continues to focus on optimizing plant‑derived anticancer strategies and enhancing their suitability for biomedical applications^5^. Cancer can arise in almost any tissue of the human body and is characterized by the loss of normal growth control, uncontrolled cell proliferation, and the potential to invade distant sites^6^. In 2022, it was responsible for approximately 9.7 million deaths worldwide, underscoring its major impact on global health^7^. Among the various malignancies, breast cancer is the most frequently diagnosed cancer in women and remains a leading cause of cancer‑related mortality, with more than 2 million new cases reported annually^6,7^. Despite advances in conventional modalities such as surgery, chemotherapy, radiotherapy, and targeted therapies, major challenges persist, including treatment resistance and systemic toxicity. These limitations have intensified interest in novel therapeutic approaches capable of selectively inducing apoptosis in cancer cells, particularly strategies that modulate oxidative stress–related pathways. Given the growing burden of cancer and the limitations of current therapeutic modalities, nanotechnology has emerged as a promising strategy to improve the delivery, stability, and biological performance of therapeutic agents. Among nanoscale systems, semiconductor quantum dots (QDs) have received particular attention due to their unique optical properties, high surface area, and modifiable surfaces, which collectively enhance cellular uptake and therapeutic interactions^8^. Furthermore, previous studies have demonstrated that eco‑friendly phytogenic and biopolymer‑assisted nanomaterials including plant‑mediated silver nanoparticles, green‑synthesized chitosan nanoparticles, graphene oxide–silver nanocomposites, and biopolymer‑stabilized gold nanoparticles—exhibit a broad spectrum of biological activities, such as antioxidant, antibacterial, cytotoxic, and enzyme‑modulating effects. Notably, several of these bio‑derived nanostructures have also shown promising anticancer properties in various experimental models, underscoring the biomedical relevance of sustainably synthesized nanomaterials.^9–13^. These findings collectively emphasize the value of eco‑friendly nanoplatforms and support the development of plant‑mediated strategies for fabricating functional QDs with potential biomedical and anticancer applications.

Recent studies suggest that nanomaterials such as quantum dots (QDs) may contribute to anticancer strategies by modulating intracellular oxidative stress and apoptosis‑related signaling pathways. In MCF‑7 breast cancer cells, QDs can enter the cytoplasm through endocytic uptake and subsequently trigger cellular stress responses, frequently associated with elevated reactive oxygen species (ROS) levels^14^. Excess ROS disrupts mitochondrial membrane potential and promotes the release of cytochrome c, initiating the intrinsic apoptotic cascade through caspase‑9 and caspase‑3 activation. ROS‑driven perturbations in cell cycle and survival pathways may further sensitize cancer cells to apoptosis^15^.

Beyond mitochondrial dysfunction, oxidative stress also affects transcriptional regulation of apoptosis‑ and redox‑related genes. Increased ROS can shift the balance toward pro‑apoptotic signaling by upregulating BAX and downregulating the anti‑apoptotic gene BCL‑2. At the same time, oxidative stress weakens the antioxidant defense system by altering the expression of enzymes such as SOD1, CAT, and GPX1, thereby amplifying intracellular ROS accumulation^16^. Previous studies indicate that quantum dots, as potent ROS‑generating nanomaterials, can exert anticancer activity not only through direct cytotoxicity but also by influencing key apoptosis‑related signaling pathways. However, the molecular mechanisms by which green‑synthesized quantum dots derived from medicinal plants, particularly those fabricated using *D. maritima*, regulate apoptosis‑associated gene expression in MCF‑7 breast cancer cells remain largely unclear. In particular, the potential roles of oxidative stress generation and caspase‑mediated apoptotic signaling induced by *D. maritima*‑derived QDs have not yet been systematically investigated^17^.

Conventional chemical reduction–based synthesis routes for nanoparticles often involve chemical reagents and stabilizing agents that raise concerns regarding toxicity and biocompatibility, motivating the development of greener and safer alternatives^18^. In contrast, green synthesis approaches utilizing plant extracts as natural reducing and capping agents offer a sustainable, cost‑effective, and biocompatible alternative. Phytochemicals such as phenolics, flavonoids, and saponins can facilitate controlled nucleation and stabilization of QDs under mild conditions, while simultaneously imparting intrinsic biological functionality. As a result, green‑synthesized QDs frequently exhibit enhanced antioxidant activity, reduced toxicity toward normal cells, and improved anticancer efficacy. Within this framework, *D. maritima* represents a promising yet under‑explored biological resource for the fabrication of functional quantum dots. The rich phytochemical profile of *D. maritima* bulb extracts provides an excellent platform for the eco‑friendly synthesis of bioactive QDs with potential anticancer applications^18,19^. MCF‑7 human breast adenocarcinoma cells were selected in this study as a well‑established estrogen receptor‑positive breast cancer model that is extensively used to investigate oxidative stress‑induced apoptosis and mitochondrial signaling pathways. Therefore, evaluating intracellular ROS generation, mitochondrial dysfunction, and caspase activation is crucial for elucidating the anticancer mechanisms of green‑synthesized QDs.

Accordingly, the present study aimed to develop a sustainable green synthesis strategy for fabricating silver quantum dots (Ag QDs) using the aqueous bulb extract of *D. maritima* as a natural reducing and stabilizing agent. The physicochemical characteristics of the biosynthesized Ag QDs were comprehensively investigated, followed by systematic evaluation of their antioxidant performance using multiple in vitro radical‑scavenging and redox‑based assays. In addition, preliminary biosafety assessments were conducted to confirm their biocompatibility. The anticancer potential of the green‑synthesized Ag QDs was subsequently examined in human breast adenocarcinoma (MCF‑7) cells, with particular emphasis on growth inhibition and oxidative stress‑mediated mechanisms. Special attention was directed toward investigating the potential involvement of ROS‑associated mitochondrial dysfunction, caspase‑dependent intrinsic apoptotic signaling, and the modulation of apoptosis‑ and redox‑related gene expression pathways. Overall, this study seeks to provide preliminary mechanistic insight into the anticancer activity of *D. maritima*-derived Ag QDs and to highlight their promise as a multifunctional and eco‑friendly nanotherapeutic platform for breast cancer treatment.

## Materials and methods

### Materials and instruments

All chemicals and reagents employed in this study, including AgNO₃, ethylene glycol, hydrazine hydrate, TPTZ, FeCl₃·6 H₂O, FeSO₄, AlCl₃·6 H₂O, NaC₂H₃O₂·3 H₂O, DPPH, ABTS, potassium persulfate, methanol, gallic acid, nitric oxide radical generators, and sodium carbonate were of analytical grade and obtained from Merck. The human breast cancer cell line MCF-7 was obtained from the National Cell Bank of Iran (Pasteur Institute of Iran, Tehran). Plant extracts were prepared using a microwave‑assisted system (DAEWOO, model KOC‑9N8T, Korea). Ultrasonic treatments were carried out in a temperature‑controlled ultrasonic bath (Pulse KMH1‑120W6501, Italy) operated at a frequency of 40 kHz and an output power of 120 W. Optical absorption characteristics were examined using a Perkin‑Elmer Lambda 25 UV–Vis spectrophotometer, FTIR-460 Plus spectrometer (JASCO, Japan). Microplate reader (BioTek), Confocal laser scanning microscope (Zeiss LSM 710). Structural crystallinity was evaluated by X‑ray diffraction employing a Panalytical X’Pert PRO diffractometer, while particle morphology and size were investigated through transmission electron microscopy using a Philips CM30 instrument. Fluorescence emission properties were measured with a Cary Eclipse spectrofluorometer (Varian).

### The aqueous extract preparation

Plant material of *D. maritima* used in this study was obtained from plants cultivated in the greenhouse of Shahid Beheshti University, Tehran, Iran, in May 2025, during the active growth stage. The plant material was air-dried under shade for four weeks, finely milled to obtain a uniform powder, and stored in airtight containers until further processing. For microwave-assisted extraction, 15 g of the powdered plant material was suspended in 200 mL of double-distilled water in an Erlenmeyer flask. Microwave irradiation was then applied for 15 min at a fixed power of 270 W, which was selected based on our previous optimization studies. Following irradiation, the extract was filtered to remove particulate matter and stored under refrigerated conditions until subsequent analyses^20^.

### Preparation of Ag QDs

Ag-QDs were synthesized through an environmentally benign green synthesis approach using *D. maritima* bulb extract as a natural reducing and stabilizing agent, following a protocol consistent with previously reported plant‑mediated Ag‑QD fabrication methods^21^. Briefly, an aqueous solution of silver nitrate (AgNO₃, 1.0 mM) was freshly prepared using deionized water. For the biosynthesis process, 50 mL of the AgNO₃ solution was mixed with 25 mL of the *D. maritima* bulb extract under ultrasonic agitation. The reaction mixture was then subjected to ultrasonic irradiation at 45 °C for 20 min to enhance nucleation and uniform growth of Ag‑QDs. To minimize undesired oxidation and ensure controlled particle formation, the synthesis was carried out under a nitrogen atmosphere. Following completion of the reaction, an equal volume of 50% (v/v) ethanol was added to the mixture to induce precipitation of unreacted biomolecules and excess phytochemical residues. The resulting suspension was centrifuged at 4500 rpm for 15 min using a microprocessor‑controlled centrifuge. After centrifugation, the clear supernatant containing well‑dispersed Ag‑QDs was carefully collected and stored at 4 °C for further physicochemical characterization and biological evaluations.

### Quantitative assessment of phenolic and flavonoid constituents

The total phenolic content of the samples (extract and Ag QDs) was quantified using the Folin–Ciocalteu colorimetric assay, following a modified protocol previously described in the literature. Quantification of phenolic constituents was carried out by measuring the absorbance at a wavelength of 765 nm, followed by calculation of their concentrations using a gallic acid‑based calibration curve. The obtained values were reported as milligrams of gallic acid equivalents per gram of sample (mg GAE/g DW)^22^.

Total flavonoid content was assessed by a spectrophotometric method based on aluminum chloride (AlCl₃) complex formation, in accordance with established procedures. Absorbance was measured at 510 nm, and flavonoid concentrations were calculated using a standard curve prepared with rutin. The final values were reported as micrograms of rutin equivalents per gram of sample (mg Rutin/g DW)^23^.

### Assessment of antioxidant performance

In this study, we examined the antioxidant performance of both the plant extracts and their biosynthesized QDs using a range of complementary in vitro assays. This approach allowed us to capture different aspects of their antioxidant behavior across multiple mechanistic pathways. The assessment panel included DPPH for free‑radical scavenging, ABTS expressed as Trolox equivalent antioxidant capacity (TEAC), FRAP for ferric‑reducing power, nitric oxide (NO) scavenging as a biologically relevant reactive species, and metal‑ion chelating activity.

### DPPH method

The antioxidant capacity of the investigated samples was evaluated based on their ability to neutralize the 1,1‑diphenyl‑2‑picrylhydrazyl (DPPH) free radical. Absorbance of both the samples and the control was measured at 517 nm using a UV–visible spectrophotometer. The radical scavenging activity was expressed as the percentage of inhibition.

1 $$\:Inhibition\left(\%\right)=\:\left[\left(\raisebox{1ex}{${A}_{0}-{A}_{1}$}\!\left/\:\!\raisebox{-1ex}{${A}_{0}$}\right.\right)\times\:100\right]$$

where A_0_ and A_1_ correspond to the absorbance values recorded for the control and the sample, respectively, respectively^24^.

### Assessment of antioxidant capacity using the ABTS assay

To further characterize the antioxidant potential of the tested samples, an ABTS radical cation decolorization assay was applied following a slightly modified version of the procedure originally proposed by Arnao. The ABTS•⁺ radical cation was generated by reacting 2,2′‑azinobis(3‑ethylbenzothiazoline‑6‑sulfonic acid) with potassium persulfate. The ability of the samples to scavenge this radical was quantified by measuring absorbance at 734 nm using a UV–Vis spectrophotometer. The percentage of radical inhibition was calculated using the same model applied in the DPPH assay. Trolox, a synthetic antioxidant analog of vitamin E, was used as the reference standard in both the ABTS and DPPH assays^25^.

### Assessment of ferric ion–reducing capacity

The reducing ability of the samples was assessed by the ferric reducing antioxidant power (FRAP) method, which is based on the conversion of ferric (Fe³⁺) to ferrous (Fe²⁺) ions under acidic conditions. Quantification was performed by comparing sample responses with a calibration curve constructed using ferrous sulfate (FeSO₄) solutions in the concentration range of 100–1000 µM. The final results were expressed as micromoles of Fe²⁺ equivalents per gram of the tested sample^26^.

### NO index

Nitric oxide radical scavenging activity of the samples was assessed using a spectrophotometric method derived from the Griess–Illosvoy reaction, following the protocol reported by Adeleke et al. (2024)^27^. In this procedure, various concentrations of the test materials were allowed to react with sodium nitroprusside at 25 °C for 180 min, enabling the controlled release of nitric oxide. After the incubation period, freshly prepared Griess reagent was added in equal volume to each reaction mixture. The resulting chromophore was quantified by measuring absorbance at 546 nm. The nitric oxide radical scavenging activity was calculated by comparing the absorbance values of treated samples with those of the untreated control, using a formula analogous to that employed in the DPPH assay Eq. (1).

### Metal chelating potential assay

The ferrous ion chelating capacity of the samples was determined following a modified protocol adapted from previous reports. Briefly, 1 mL aliquots of the samples (1.0 mg/mL) were mixed with 1.0 mL of methanol followed by the addition of 100 µL of FeCl₂ solution (2.0 mM). The mixtures were allowed to stand at ambient temperature for 10 min. Chelation was then initiated by adding 200 µL of freshly prepared ferrozine solution (5.0 mM), and the reaction mixtures were incubated at 25 °C for an additional 10 min. The resulting Fe²⁺–ferrozine complexes were quantified by measuring absorbance at 562 nm using a UV–visible spectrophotometer. Ferrous ion chelating activity was expressed as percentage inhibition relative to the control, using a calculation analogous to that employed in the DPPH assay (Eq. 1), with disodium ethylenediaminetetraacetate (EDTA) serving as the reference chelator^28^.

### MTT cell viability assay

The metabolic activity of MCF‑7 cancer cells and MCF‑10 A normal breast epithelial cells (used as the non‑cancerous control cell line) was assessed using the MTT colorimetric assay, which is based on the ability of mitochondrial dehydrogenase enzymes in viable cells to convert 3‑(4,5‑dimethylthiazol‑2‑yl)-2,5‑diphenyltetrazolium bromide (MTT) into insoluble purple formazan crystals. Briefly, both cell lines were enzymatically detached, collected, and seeded into 96‑well plates at a density of 1.4 × 10⁴ cells per well in 200 µL of complete culture medium. The plates were incubated for 48 h at 37 °C in a humidified atmosphere with 5% CO₂ to allow proper cell attachment and stabilization prior to treatment.

Following incubation, the cells were exposed to various concentrations of the test samples within the range of 1.95–4000 µg/mL under conventional static culture conditions for the designated exposure period. At the end of the treatment, the culture medium was gently removed and replaced with 200 µL of MTT solution (0.5 mg/mL in phosphate‑buffered saline, PBS). The plates were then incubated for 3 h at 37 °C to facilitate the enzymatic reduction of MTT to formazan by metabolically active cells. Subsequently, the supernatant was carefully aspirated, and 100 µL of dimethyl sulfoxide (DMSO) was added to each well to dissolve the formazan crystals under mild shaking at 37 °C. Absorbance was recorded at 570 nm using a microplate ELISA reader, and cell viability (%) was calculated according to Eq. (2):

2 $$Cell\;viability\;(\% )=\frac{{{\mathrm{Treated}}\;{\mathrm{culture}}\;{\mathrm{absorbance}}}}{{{\mathrm{Untreated}}\;{\mathrm{culture}}\;{\mathrm{absorbance}}}} \times 100$$

The resulting dose–response curves were then used to determine IC₅₀ values. All experiments were performed in triplicate to ensure reproducibility and statistical reliability^29^.

### Assessment of intracellular ROS production

Intracellular oxidative stress in MCF‑7 human breast carcinoma cells was evaluated by measuring reactive oxygen species (ROS) generation using the fluorogenic probe 2′,7′‑dichlorodihydrofluorescein diacetate (DCFH‑DA). After cellular uptake, DCFH‑DA was deacetylated by intracellular esterases to produce the non‑fluorescent compound 2′,7′‑dichlorodihydrofluorescein, which was subsequently oxidized by ROS to form the highly fluorescent 2′,7′‑dichlorofluorescein (DCF). Therefore, intracellular ROS levels were estimated based on the intensity of the emitted fluorescence. MCF‑7 cells were cultured in RPMI‑1640 medium supplemented with 10% fetal bovine serum and 1% penicillin–streptomycin, and maintained at 37 °C in a humidified atmosphere containing 5% CO₂. For the experiment, 1 × 10⁴ cells were seeded into black 96‑well microplates with transparent bottoms and incubated for 24 h to allow cell attachment. The cells were then treated for an additional 24 h with increasing concentrations of biosynthesized Ag QDs, including doses below and above the IC₅₀ value, while untreated wells served as the control group. Following treatment, the culture medium was removed and the cells were washed twice with ice‑cold phosphate‑buffered saline (PBS) to eliminate residual compounds. Cells were then detached using trypsin–EDTA, collected by mild centrifugation, and washed twice to remove remaining enzymes and extracellular nanoparticles. The resulting cell pellets were resuspended in PBS or serum‑free medium and incubated with freshly prepared DCFH‑DA (10 µM) for 30 min at 37 °C under dark conditions. After removing excess dye, fresh serum‑free medium was added and fluorescence was measured. DCF fluorescence was quantified using a microplate reader at an excitation wavelength of 485 nm and an emission wavelength of 520 nm, and the results were expressed as mean fluorescence intensity (MFI). Cells treated with hydrogen peroxide (100 µM) were used as the positive control^30^.

### Flow cytometry analysis

Apoptosis was evaluated using Annexin V‑FITC/propidium iodide (PI) staining followed by flow cytometry. Cells were harvested, washed with phosphate‑buffered saline (PBS), and centrifuged at 1500 rpm for 5 min. The resulting cell pellet was resuspended in 500 µL of 1× Binding buffer. To ensure proper fluorescence compensation and to prevent signal overlap between fluorescein isothiocyanate (FITC) and propidium iodide (PI), the cell suspension was divided into four separate aliquots: an unstained control, cells stained with Annexin V‑FITC alone, PI‑only stained cells, and cells subjected to dual staining with Annexin V‑FITC and PI. The unstained and PI‑only samples were maintained at 4 °C throughout the staining procedure.

Next, 3 µL of Annexin V‑FITC was dispensed into the designated tubes, after which the samples were kept for 15 min at 4 °C under light‑protected conditions. Once the incubation period was complete, each tube received 1 mL of 1× Binding Buffer, and the cells were pelleted by centrifugation at 1500 rpm for 5 min. The supernatant was then gently removed, and the cell pellets were resuspended in 500 µL of freshly prepared 1× Binding Buffer. Just before acquisition on the flow cytometer, 3 µL of propidium iodide was added to the specified samples.

Data acquisition was performed using standard flow cytometry settings, with fluorescence signals corresponding to Annexin V‑FITC and PI collected at emission wavelengths of 520 nm and 617 nm, respectively, in accordance with the manufacturer’s instructions and established protocols^31^.

### Assessment of caspase‑3/9 activity levels

Caspase‑3 and caspase‑9 activities were quantified using luminescent assay kits (Caspase‑Glo‑3 and Caspase‑Glo‑9, Promega, USA) following the manufacturer’s instructions. MCF‑7 cells were seeded into 96‑well plates at a density of 5 × 10⁴ cells per well and incubated for 24 h at 37 °C in a humidified 5% CO₂ atmosphere to allow cell attachment. The cells were then treated with Ag quantum dots at their IC₅₀ concentration for 24 h, while untreated wells served as negative controls.

Before measurement, the plates were equilibrated to room temperature. Caspase‑Glo reagent (100 µL) was added to each well containing 100 µL of culture medium, gently mixed for 30 s at 500 rpm, and incubated at room temperature for 30 min.

Luminescence was recorded using a microplate reader, and the resulting values were normalized to those of untreated control cells, which were defined as a relative activity of 1.0^32^.

### Confocal laser scanning microscopy (CLSM) imaging

Cellular uptake and intracellular localization of Ag QDs were evaluated using confocal laser scanning microscopy. MCF‑7 breast cancer cells were seeded on sterile glass coverslips placed in 6‑well plates and cultured until reaching approximately 70–80% confluency. The cells were then treated with Ag QDs at a sub‑cytotoxic concentration (½ IC₅₀) for 24 h to ensure preservation of cellular morphology, while untreated cells served as negative controls.

After incubation, cells were washed with PBS and fixed with 4% paraformaldehyde for 15 min at room temperature. Membrane permeabilization was performed using 0.1% Triton X‑100, followed by nuclear counterstaining with DAPI (5 µg/mL) for 10 min in the dark. The coverslips were mounted using an anti‑fade reagent and imaged with a confocal laser scanning microscope.

Ag QDs were excited at 488 nm with emission collected between 500 and 550 nm, while DAPI‑stained nuclei were visualized using 405 nm excitation and emission detection between 420 and 480 nm. Imaging parameters were optimized using untreated control cells, and Z‑stack sections were acquired to assess internalization. Final images and merged channels were processed using ZEN software to confirm intracellular uptake of Ag QDs at non‑lethal exposure levels^33^.

### Transcriptional and mitochondrial analyses of apoptosis-related mechanisms

#### RNA extraction and cDNA synthesis

Total RNA was extracted from MCF‑7 human breast adenocarcinoma cells (approximately 1 × 10⁶ cells per sample) following 24 h exposure to green‑synthesized Ag QDs at different concentrations (0, 25, 50, and 100 µg/mL). RNA isolation was performed using TRIzol^®^ reagent (Invitrogen, USA) in accordance with the manufacturer’s instructions. The quantity and purity of the isolated RNA were evaluated spectrophotometrically using a NanoDrop™ 2000 instrument (Thermo Scientific, USA) by assessing the A₂₆₀/A₂₈₀ (1.8–2.0) and A₂₆₀/A₂₃₀ (> 2.0) absorbance ratios. RNA integrity was further confirmed by electrophoresis on a 1% agarose gel.

For complementary DNA (cDNA) synthesis, 1 µg of total RNA was reverse‑transcribed using the PrimeScript™ RT reagent Kit (Takara Bio, Japan) with random hexamer primers in a final reaction volume of 20 µL. The reverse‑transcription program consisted of incubation at 37 °C for 15 min, followed by enzyme inactivation at 85 °C for 5 s. The synthesized cDNA was diluted ten‑fold with nuclease‑free water and stored at − 20 °C until subsequent qRT‑PCR analysis^34^.

#### Primer design and validation

Gene‑specific primers targeting key apoptosis‑ and oxidative stress‑related genes were designed using Primer‑BLAST (NCBI) and synthesized commercially by Macrogen (Seoul, Korea). The selected genes included pro‑apoptotic markers (BAX, CASP9, CASP3), the anti‑apoptotic gene BCL‑2, and antioxidant defense genes (SOD1, CAT, and GPX1). GAPDH was used as the internal reference gene to normalize expression levels.

Primer specificity was verified by melt‑curve analysis, which consistently showed a single sharp peak for each amplicon, as well as by agarose‑gel electrophoresis of PCR products. Amplification efficiency and linearity were assessed using a 10‑fold serial dilution of pooled cDNA samples (10⁰–10⁻⁴). All primer pairs exhibited acceptable amplification efficiencies ranging from 95% to 105%, with correlation coefficients (R²) ≥ 0.998, indicating high reliability and optimal qPCR performance.

#### Quantitative real‑time PCR amplification

Quantitative real‑time PCR was carried out using SYBR^®^ Green Premix Ex Taq™ (Takara Bio, Japan) on a StepOnePlus™ Real‑Time PCR System (Applied Biosystems, USA). Each 20 µL reaction mixture consisted of 10 µL SYBR Green master mix, 0.5 µM of each forward and reverse primer, 2 µL of diluted cDNA, and nuclease‑free water.

The thermal cycling conditions included an initial denaturation step at 95 °C for 30 s, followed by 40 amplification cycles of denaturation at 95 °C for 5 s and annealing/extension at 60 °C for 30 s. A melt‑curve analysis was performed at the end of each run (95 °C for 15 s, 60 °C for 1 min, followed by a gradual increase to 95 °C at 0.3 °C/s) to confirm amplification specificity. Non‑template controls (NTC) and no‑reverse‑transcription controls (NRT) were included in all experiments to exclude contamination and genomic DNA interference^34^. All reactions were performed in technical triplicate.

#### Assessment of mitochondrial membrane potential (ΔΨm)

Alterations in mitochondrial membrane potential (ΔΨm) in MCF‑7 cells following exposure to green‑synthesized Ag QDs were evaluated using the JC‑1 fluorescent probe, a well‑established indicator of mitochondrial depolarization during intrinsic apoptosis. Briefly, MCF‑7 cells were seeded into 6‑well plates at a density of 2 × 10⁵ cells per well and incubated under standard culture conditions (37 °C, 5% CO₂) for 24 h to allow stabilization. Cells were then treated with Ag QDs at the IC₅₀ concentration for an additional 24 h. Untreated cells served as the negative control, whereas cells exposed to carbonyl cyanide m‑chlorophenyl hydrazone (CCCP, 50 µM for 30 min) were used as a positive control for mitochondrial depolarization.

Following treatment, cells were harvested by gentle centrifugation (1500 rpm, 5 min), washed twice with phosphate‑buffered saline (PBS), and resuspended in serum‑free medium containing JC‑1 dye at a final concentration of 5 µg/mL. The cells were incubated at 37 °C for 20 min in the dark to allow dye accumulation within mitochondria. Excess dye was removed by washing twice with PBS.

Fluorescence signals corresponding to JC‑1 monomers (green fluorescence) and JC‑1 aggregates (red fluorescence) were measured using a fluorescence microplate reader or flow cytometer at excitation/emission wavelengths of 485/530 nm and 540/590 nm, respectively^35^. Mitochondrial membrane potential was expressed as the ratio of red to green fluorescence intensity. A decrease in this ratio was interpreted as mitochondrial depolarization and loss of ΔΨm. All experiments were conducted in triplicate (*n* = 3), and results were reported as mean ± standard deviation (SD).

#### Gene‑expression data analysis

Threshold cycle (Ct) values were obtained using StepOne™ software v2.3 (Applied Biosystems). Relative gene expression levels were calculated using the comparative 2⁻ΔΔCt method as described by Livak and Schmittgen (2001). Briefly, ΔCt values were calculated as Ct(target gene) – Ct(GAPDH), and ΔΔCt values were obtained by subtracting the ΔCt of the control group from that of the treated samples. Fold‑change values were expressed as mean ± SD of three independent biological replicates, each analyzed in technical triplicate.

### Statistical analysis

Statistical analyses were performed using SAS software version 9.4 (SAS Institute Inc., Cary, NC, USA). Data were analyzed by one‑way or factorial analysis of variance (ANOVA), as appropriate. Mean comparisons were primarily conducted using Tukey’s honestly significant difference (HSD) post‑hoc test to control for multiple comparisons. In parallel, Least Significant Difference (LSD) analysis was applied for planned comparisons, yielding consistent grouping patterns. Results were expressed as mean ± standard error (SE), and differences were considered statistically significant at *p* < 0.05. Graphical representation and descriptive statistical analyses were carried out using OriginPro 2024 (OriginLab Corp., Northampton, MA, USA).

## Result

### Characterization of the synthesized Ag QDs

**Fig. 1:**
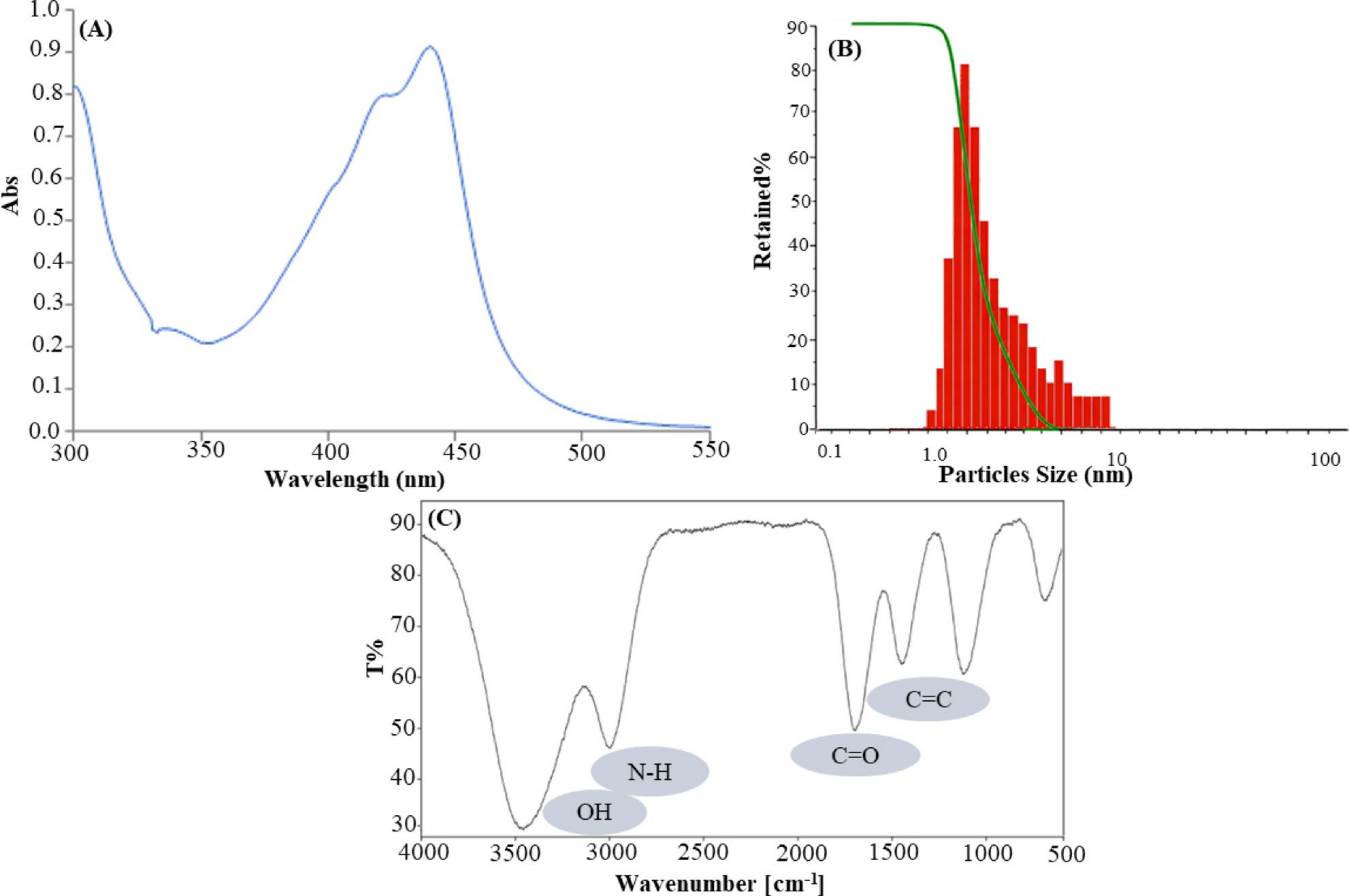
(**A**) UV–Vis absorption spectrum, (**B**) Particle size distribution of Ag QDs measured by DLS (the green line is shown as a guide to the eye) and, (**C**) FT-IR of the Ag QDs.

The UV–visible absorption spectrum (Fig. 1A) exhibited a distinct and sharp peak centered at 437 nm, corresponding to the surface plasmon resonance (SPR) of silver nanostructures, thereby verifying the successful synthesis of Ag QDs. In addition, dynamic light scattering (DLS) analysis indicated that the prepared Ag QDs displayed a mean hydrodynamic particle size of approximately 8.10 nm (Fig. 1B). The FT‑IR spectrum of the synthesized Ag QDs (Fig. 1C) exhibited several characteristic absorption bands indicative of biomolecule-assisted reduction and capping. A broad and intense band around 3200–3400 cm⁻¹ corresponded to O–H stretching vibrations, confirming the presence of hydroxyl-containing compounds adsorbed on the nanoparticle surface. A shoulder in the same region was attributed to N–H stretching of amine or amide groups originating from proteins or other nitrogenous biomolecules. The pronounced peak near ~ 1630 cm⁻¹ was assigned to C = O stretching (amide I or carbonyl groups), suggesting their involvement in both the reduction of Ag⁺ and stabilization of the formed nanoparticles. Additionally, bands in the 1500–1400 cm⁻¹ region corresponded to C = C vibrations of aromatic or unsaturated structures, while multiple peaks in the fingerprint region (1200–600 cm⁻¹) reflected C–O, C–N, and C–C skeletal vibrations of organic residues coating the Ag QDs.

Overall, the FT‑IR results confirm that hydroxyl, amine, carbonyl, and aromatic groups from the biological extract remained attached to the nanoparticle surface, acting as effective reducing and capping agents.

**Fig. 2:**
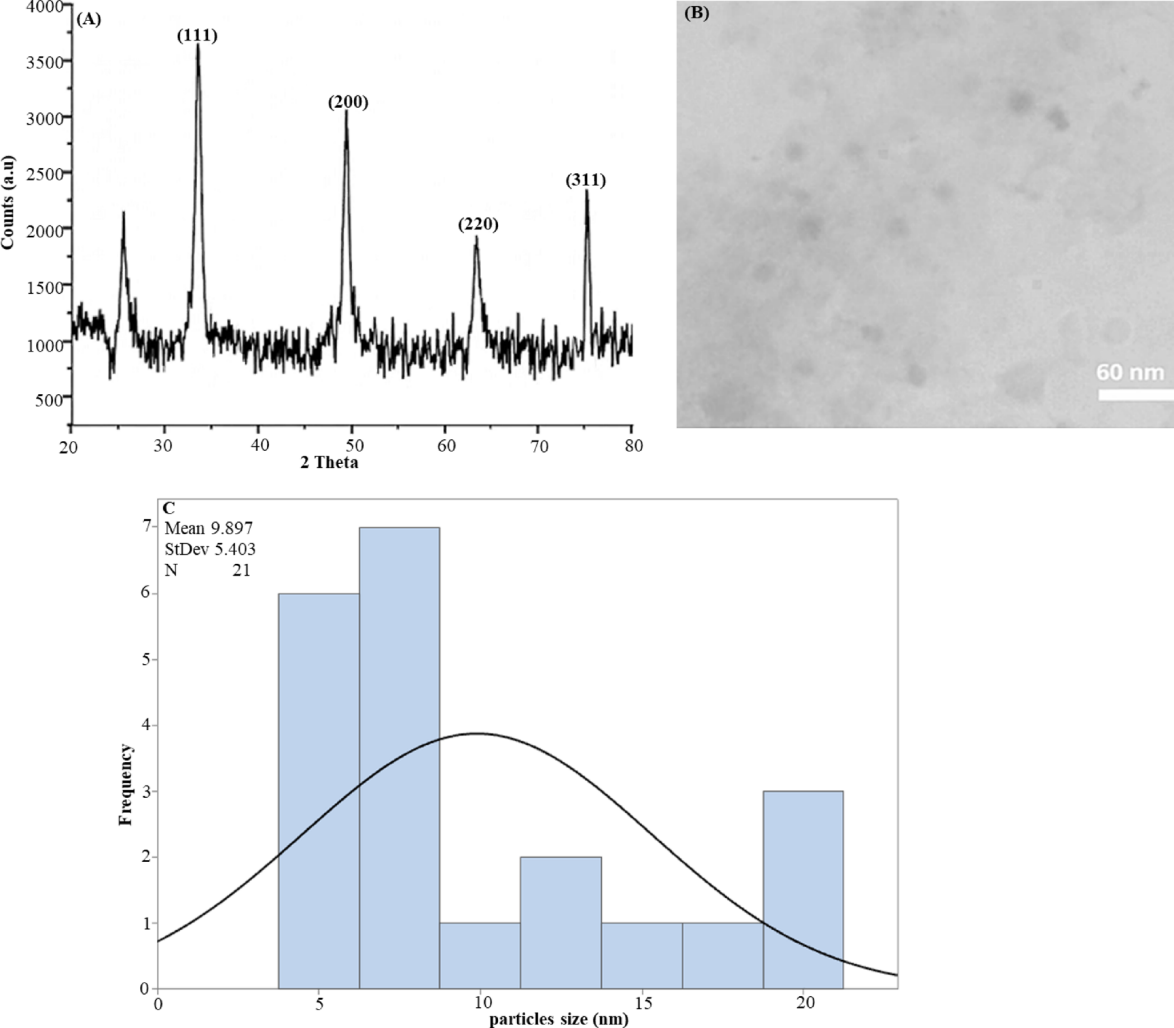
Comprehensive characterization of the Ag QDs synthesized: (**A**) XRD confirms fcc crystalline silver nanocrystals (< 10 nm); (**B**) TEM image, (**C**) Particle size distribution histogram with Gaussian fitting indicating an average diameter of ~ 9–10 nm.

Evaluation of the surface charge by zeta potential measurements revealed that the Ag quantum dots carried a net negative charge of − 27.9 mV, reflecting strong electrostatic repulsion between particles and, consequently, high colloidal stability in aqueous environments. Structural characterization using X‑ray diffraction (XRD) further verified both the crystalline nature and phase purity of the synthesized nanomaterials (Fig. 2A). The recorded diffraction pattern displayed distinct Bragg peaks attributable to the (111), (200), (220), and (311) lattice planes of face‑centered cubic (fcc) metallic silver, providing clear evidence for the formation of crystalline Ag⁰ domains. Notably, the pronounced intensity of the (111) plane indicates preferential crystal orientation along this direction, a behavior frequently reported for silver nanostructures produced via green or biologically mediated synthesis routes. The slight peak broadening further indicates nanoscale crystallite dimensions, while the weak diffuse background is attributed to residual amorphous phytochemical capping layers that contribute to colloidal stabilization. TEM analysis (Fig. 2B) revealed that the Ag QDs were predominantly spherical and relatively well dispersed, with an average particle diameter of approximately 9.8 nm. Although slight aggregation was occasionally observed, the primary nanoparticles were clearly distinguishable and remained below 10 nm in size, consistent with quantum‑dot dimensions. Overall, these observations confirm the nanoscale size and relatively uniform morphology of the synthesized Ag QDs obtained via the green synthesis approach. The particle size distribution analysis (Fig. 2C) further supports the TEM observations. The histogram indicates that the majority of particles fall within the size range of approximately 8–16 nm, with an average diameter of about 9–10 nm. The Gaussian fitting curve shows a central peak corresponding to the dominant particle population, indicating a relatively uniform size distribution with moderate variation in particle size. These results suggest that the green synthesis method enables reasonably controlled nucleation and growth of Ag QDs.

### Biological properties

#### Analysis of phenolic and flavonoid levels along with antioxidant capacity evaluation

The extract was found to contain substantial amounts of phenolic and flavonoid compounds, reaching approximately 165 mg GAE g/DW and 125 mg Rutin g/DW, respectively. Such elevated concentrations underscore the role of microwave irradiation in efficiently promoting the release of bioactive phytochemicals by accelerating cellular matrix disruption and improving solvent accessibility. Biologically, the abundance of these antioxidant‑associated metabolites establishes a robust biochemical basis for the enhanced antioxidant performance later recorded for both the plant extract and the synthesized Ag QDs^36,37^.

#### Antioxidant and radical scavenging performance

**Fig. 3:**
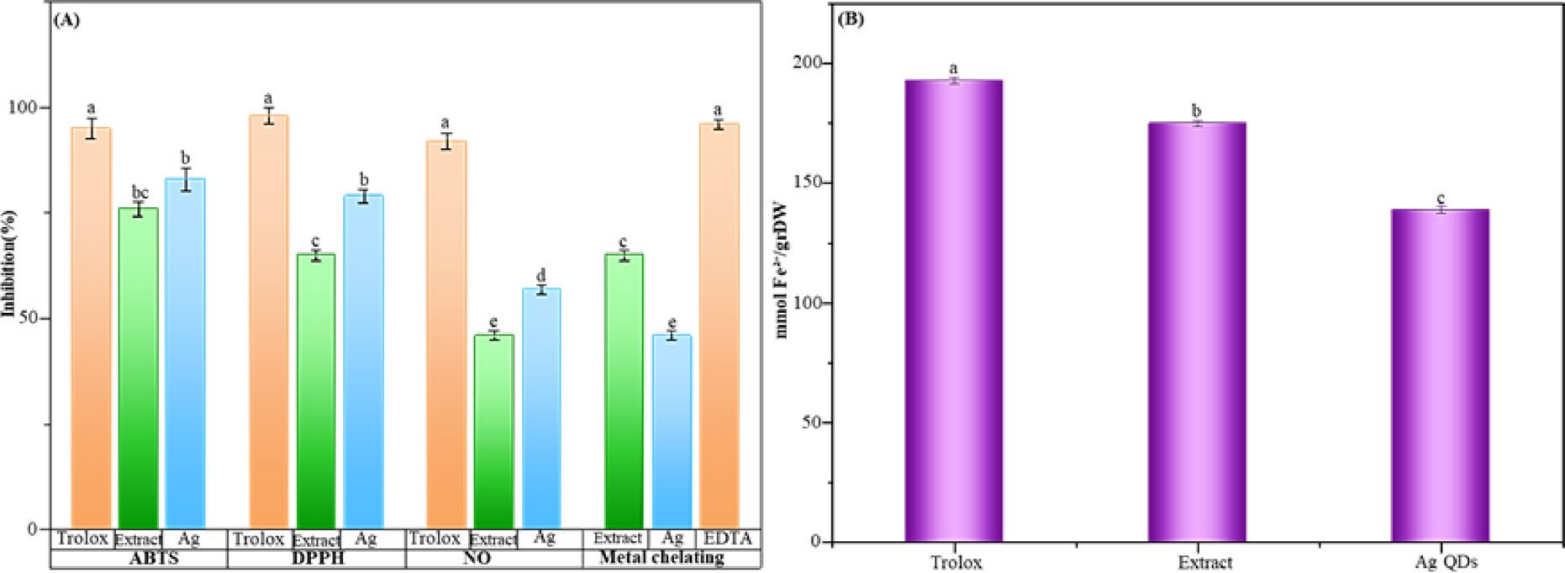
Comparative evaluation of antioxidant and biological activities of the plant extract and biosynthesized Ag QDs. (**A**) Radical‑scavenging activity assessed by DPPH, ABTS, NO, and metal‑chelating capacity and (**B**) FRAP. Data are presented as mean ± SD of triplicate experiments (*n* = 3).

The antioxidant potential of the synthesized Ag QDs was systematically evaluated using multiple complementary in vitro assays, including ABTS, DPPH, nitric oxide (NO) scavenging, and metal chelating activity, with Trolox and crude plant extract serving as reference standards (Fig. 3A). In the ABTS radical scavenging assay, Ag QDs exhibited a high inhibition efficiency of approximately 83%, outperforming the corresponding plant extract (76%) while remaining slightly lower than the Trolox standard (95%). This result indicates that nanoscale silver functionalized with phytochemical moieties retains substantial radical quenching capacity, likely arising from synergistic interactions between the metallic core and surface-bound bioactive compounds.

Similarly, in the DPPH assay, Ag QDs demonstrated strong free radical scavenging activity (≈ 79%), markedly higher than that of the extract alone (65%) and approaching the efficacy of Trolox (98%). The enhanced performance of Ag‑QDs compared to the crude extract can be attributed to the increased surface area, improved electron transfer capability, and effective stabilization of antioxidant functional groups on the nanoparticle surface.

The NO scavenging assay revealed a moderate yet significant inhibitory activity for Ag‑QDs (≈ 57%), exceeding that of the plant extract (46%) but remaining below the Trolox control (92%). This trend suggests that while Ag‑QDs are effective scavengers of reactive nitrogen species, their activity is somewhat selective and assay-dependent, as commonly reported for green-synthesized nanomaterials^21^.

In contrast, the metal chelating assay showed comparatively lower chelation efficiency for Ag‑QDs (≈ 46%) relative to the extract (65%) and the control (96%). This observation implies that the availability of metal-binding functional groups may be partially reduced upon nanoparticle formation, possibly due to their involvement in silver ion reduction and nanoparticle stabilization. Nonetheless, the preserved chelating activity confirms the presence of residual active ligands on the Ag QD surface. Overall, the antioxidant profile of Ag‑QDs was superior to that of the crude extract in the majority of radical‑scavenging assays, demonstrating that green nanostructuring significantly enhances bioactivity. The multifunctional antioxidant behavior of Ag QDs arises from the combined effects of the metallic core, nanoscale dimensions, and surface-bound phytochemicals, supporting their potential application in biomedical and pharmaceutical systems where oxidative stress mitigation is required.

The FRAP assay was employed to quantitatively evaluate the electron‑donating and reducing capacity of the samples. As shown in Fig. 3B, Trolox exhibited the highest FRAP value (193 mmol Fe²⁺ equivalents), confirming its strong and well‑established reducing potential. The plant extract displayed substantial ferric reducing activity (175 mmol Fe²⁺ equivalents), reflecting the presence of redox‑active phytochemicals capable of donating electrons and reducing Fe³⁺ to Fe²⁺. In comparison, the Ag‑QDs demonstrated a moderate reducing capacity (143 mmol Fe²⁺ equivalents), which is consistent with the partial retention of phytochemical moieties on the nanoparticle surface that contribute to electron transfer and redox activity.

Notably, the synthesized Ag QDs demonstrated a pronounced ferric reducing capacity, with a FRAP value of 139 mmol Fe²⁺ equivalents. Although this value was lower than those of Trolox and the crude extract, it remains significant and highlights the preserved redox functionality following nanoparticle formation. The slight reduction in FRAP activity relative to the extract can be attributed to partial involvement of antioxidant functional groups in silver ion reduction and nanoparticle stabilization, which may limit their direct participation in Fe³⁺ reduction.

Importantly, the observed FRAP activity of Ag QDs underscores the dominant contribution of surface‑associated phytochemicals, potentially facilitated by nanoscale interfacial effects. The nanoscale dimensions and high surface area of the Ag‑QDs facilitate effective redox interactions, supporting their function as moderate yet stable reductants under assay conditions. Overall, these findings confirm that green‑synthesized Ag‑QDs retain appreciable ferric reducing power, reinforcing their multifunctional antioxidant profile and corroborating the results obtained from radical scavenging and metal chelation assays.

Overall, all evaluated samples displayed pronounced antioxidant capacity across a comprehensive set of complementary assays, confirming their effectiveness in neutralizing diverse reactive species. Notably, Ag QDs outperformed the crude extract in radical‑quenching assays, including DPPH, ABTS, and nitric oxide scavenging. In contrast, the crude extract exhibited stronger ferric‑reducing ability and metal‑chelating activity. This dual antioxidant behavior likely arises from the redistribution and differential availability of phytochemical constituents during nanoparticle formation. Specifically, metabolites anchored to the nanoparticle surface preferentially mediate interfacial electron‑transfer reactions, whereas freely dissolved bioactives remain more proficient in bulk redox processes and metal ion sequestration^38^.

Collectively, these results highlight the pharmacological relevance of both the native extract and its Ag QDs formulation, These findings suggest that the antioxidant activity of the biosynthesized Ag‑QDs may have potential relevance in mitigating oxidative stress–related disorders, including degenerative conditions, metabolic dysfunctions such as diabetes, and neuroinflammatory diseases, warranting further in vivo investigation^39–41^. Moreover, the substantial nitric oxide scavenging activity further substantiates the anti‑inflammatory potential of *D. maritima* extracts and their nano‑engineered derivatives, a property that is consistent with their enrichment in phenolic and flavonoid compounds^42,43^.

### Anticancer activity

**Fig. 4:**
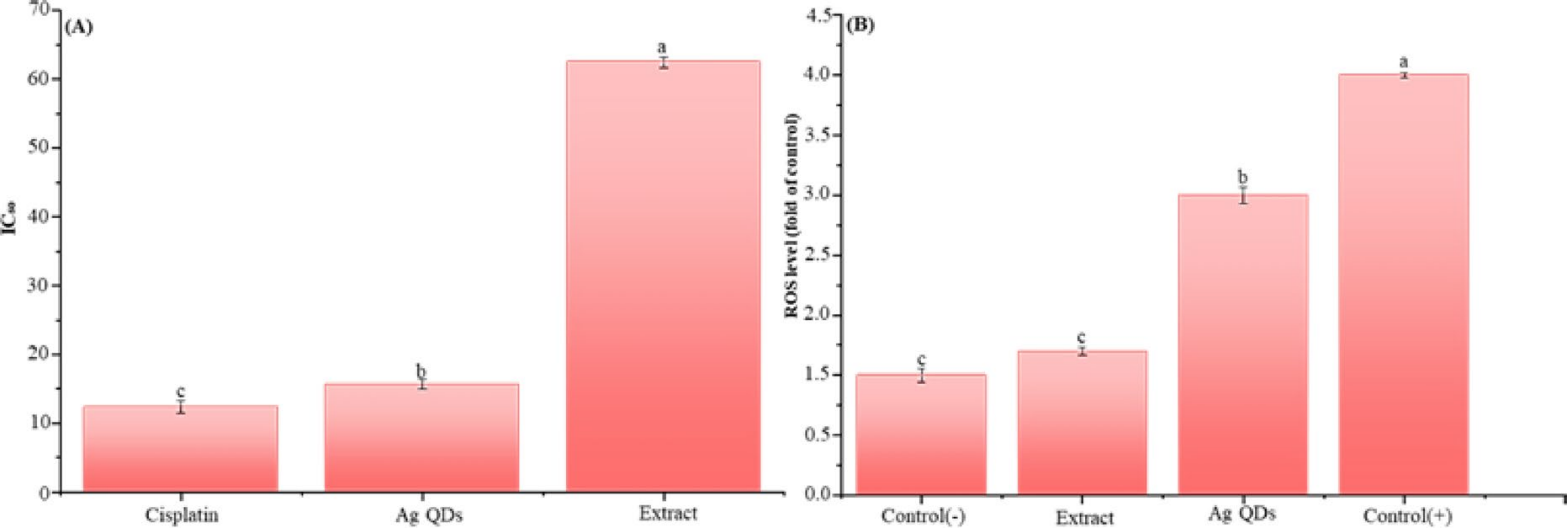
Effects of different treatments on cellular responses in MCF‑7 cells. (**A**) Cell viability assessed by the MTT assay. (**B**) Intracellular reactive oxygen species (ROS) levels. Data are presented as mean ± standard deviation (SD).

The cytotoxic effect of the biosynthesized Ag QDs was evaluated using the MTT assay and compared with the corresponding plant extract, while cisplatin was employed as a standard chemotherapeutic control. As shown in Fig. 4A, cisplatin exhibited the lowest IC₅₀ value (12.4 ± 0.96 µg/mL), confirming its well‑known cytotoxic potency and validating the reliability of the experimental conditions. Notably, the Ag QDs demonstrated pronounced growth‑inhibitory activity against MCF‑7 breast cancer cells with an IC₅₀ value of 14.70 ± 0.68 µg/mL, which was close to that of cisplatin. This comparable potency highlights the strong anticancer potential of the nanostructured system and indicates that phytochemical‑capped silver nanostructures can effectively suppress cancer cell proliferation.

The remarkable cytotoxic response induced by Ag QDs can be attributed to their nanoscale dimensions and large surface‑to‑volume ratio, which facilitate enhanced cellular internalization and stronger intracellular interactions. These properties may promote excessive reactive oxygen species (ROS) generation, mitochondrial dysfunction, and activation of apoptosis‑related signaling pathways. In contrast, the crude plant extract exhibited moderate cytotoxicity with a significantly higher IC₅₀ value (62.5 ± 0.79 µg/mL), likely due to the limited cellular uptake and lower bioavailability of phytochemical constituents in their free molecular form.

To further understand the safety of the tested materials, their cytotoxicity was also examined in the normal breast epithelial cell line MCF‑10 A. As expected, both samples showed considerably lower toxicity toward normal cells. The Ag QDs exhibited an estimated IC₅₀ of about 85 µg/mL, while the plant extract showed an even higher IC₅₀ of roughly 210 µg/mL. This difference in sensitivity suggests a degree of selective cytotoxicity toward cancer cells, while sparing non‑cancerous cells to a greater extent.

Taken together, the noticeable improvement in cytotoxic activity after the formation of Ag QDs highlights how nanostructuring can substantially enhance biological performance. This enhanced effect is likely due to more efficient cellular uptake, longer intracellular retention, and the synergistic contribution of the silver core with its phytochemical coating. Overall, these findings indicate that the green‑synthesized Ag QDs provide strong anticancer activity approaching that of cisplatin, but with better selectivity toward malignant cells. This combination of potency and selectivity positions them as promising candidates for further investigation as nano‑therapeutic agents.

Oxidative stress resulting from excessive generation of ROS in response to external stimuli is recognized as one of the primary triggers of apoptosis in cancer cells. Accumulating evidence has demonstrated that Ag QDs can induce pronounced oxidative stress, leading to the disruption of cellular redox homeostasis, mitochondrial membrane potential collapse, and enhanced lipid peroxidation, ultimately culminating in apoptotic cell death^14^.

As shown in Fig. 4B, intracellular ROS levels in MCF‑7 cells were markedly modulated by different treatments. Untreated control cells exhibited a basal ROS level normalized to 1.0, reflecting physiological redox homeostasis. Exposure to the *D. maritima* extract resulted in a modest but measurable increase in ROS production (≈ 1.2‑fold), suggesting a mild oxidative response that remains within a tolerable cellular range and is likely counterbalanced by the intrinsic antioxidant constituents of the extract. In contrast, treatment with Ag QDs (at IC_50_ concentration) led to a pronounced elevation in intracellular ROS, reaching approximately 2.5‑fold relative to the untreated control (Fig. 4B). This substantial increase indicates that nanoscale silver effectively amplifies oxidative stress, potentially through enhanced cellular internalization, surface‑mediated redox reactions, and mitochondrial perturbation. Notably, the ROS level induced by Ag QDs approached that observed in the positive control [Control +, ≈ 3.5‑fold], underscoring the strong pro‑oxidant capacity of the nano‑formulation. The differential ROS profiles between Ag QDs and the crude extract highlight the critical impact of nanostructuring on biological activity. While the extract alone triggered only a limited oxidative response, conversion into Ag QDs significantly intensified ROS generation, thereby shifting the intracellular redox balance toward oxidative stress. This ROS accumulation is of mechanistic relevance, as elevated ROS levels are well‑established upstream signals for mitochondrial dysfunction and activation of the intrinsic apoptotic pathway. Accordingly, the enhanced ROS production induced by Ag QDs provides a robust mechanistic link to the subsequent caspase‑9 and caspase‑3 activation observed in this study, supporting ROS‑mediated apoptosis as a central contributor to Ag QDs‑induced cytotoxicity in MCF‑7 cells.

**Fig. 5:**
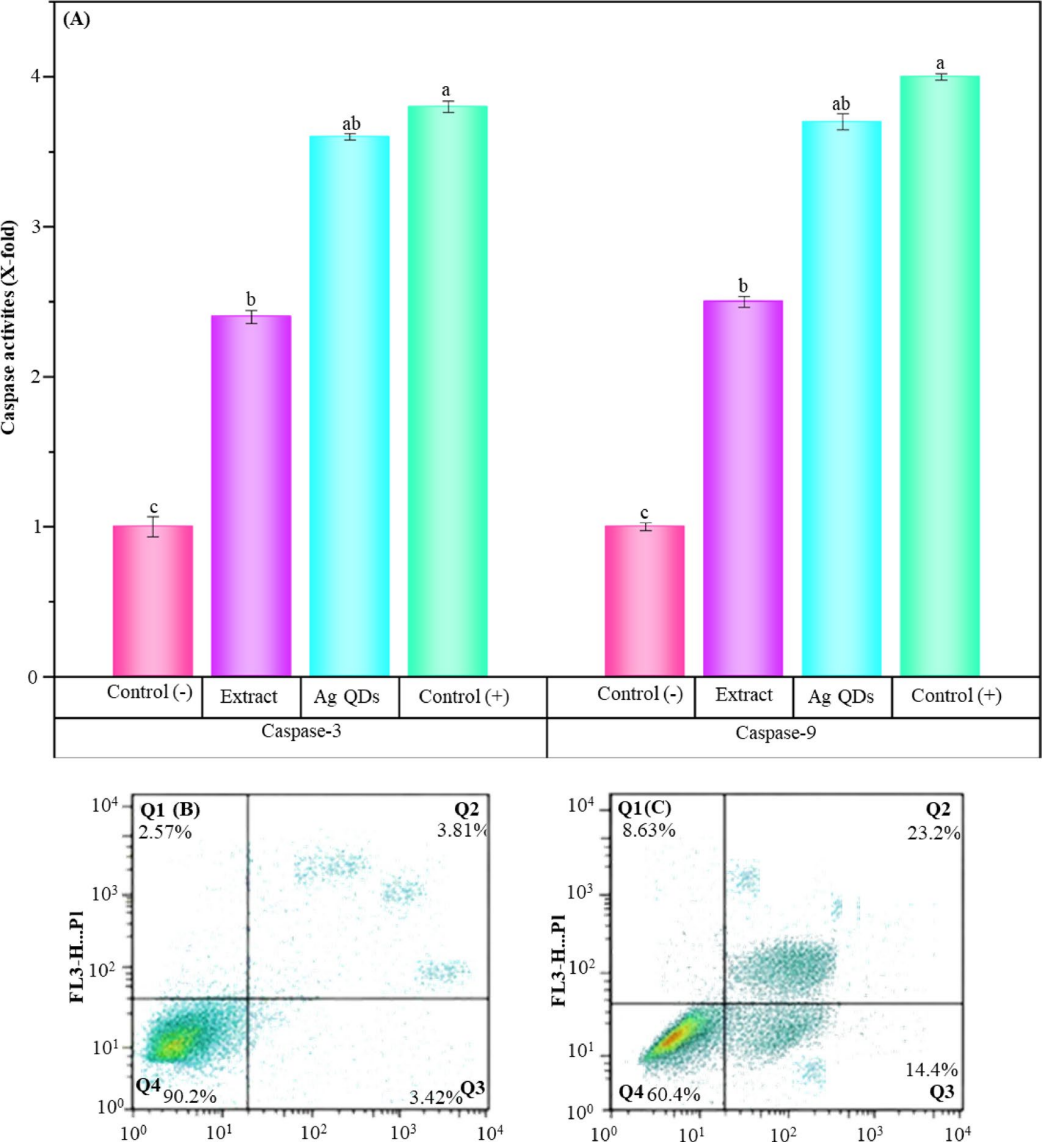
Caspase‑3 activity and caspase‑9 activity in treated and control cells (**A**). Data are expressed as fold change relative to untreated control cells (set to 1.0). Flow cytometry analysis images of untreated MCF-7 cells (**B**), and treated with Ag QDs at IC_50_ (**C**).

Figure 5A a pronounced activation of caspase‑9 was observed following treatment, indicating a dominant involvement of the intrinsic apoptotic pathway. Basal caspase‑9 activity was maintained in control cells, whereas exposure to the crude extract resulted in a clear elevation of caspase‑9 levels, reaching approximately a 2.5‑fold increase. Notably, treatment with Ag QDs led to a markedly stronger response, inducing caspase‑9 activity by nearly 3.7‑fold, which closely approached that of the positive control (4.0‑fold). This robust activation highlights the superior ability of the nano‑formulation to initiate mitochondrial‑mediated apoptotic signaling. Consistent with upstream caspase‑9 activation, a parallel increase in caspase‑3 activity was detected. The extract induced a moderate activation of caspase‑3 (2.4‑fold), while Ag QDs elicited a substantially higher response (3.6‑fold), comparable to the positive control (3.8‑fold). Importantly, within each treatment group, caspase‑9 activation exceeded that of caspase‑3, supporting a hierarchical caspase activation pattern in which mitochondrial dysfunction precedes executioner caspase activation. Collectively, these results demonstrate that Ag QDs exhibit a significantly enhanced pro‑apoptotic potential compared with the crude extract and efficiently trigger the caspase cascade via a ROS‑associated intrinsic apoptotic mechanism.

In view of the pronounced cytotoxic response observed in the MTT assay, flow cytometric analysis was employed to elucidate whether Ag QDs–induced cell death in MCF-7 cells occurred through apoptotic mechanisms. The induction of apoptosis was evaluated by dual labeling with Annexin V–FITC and propidium iodide, a strategy that enables clear differentiation among living cells as well as cells undergoing early apoptosis, late apoptosis, or necrotic death.

As illustrated in Fig. 5B, untreated MCF-7 cells predominantly localized within the viable quadrant (Q4), accounting for approximately 90.2% of the total cell population, indicative of preserved membrane integrity and the absence of apoptosis. Only minor fractions of cells were detected in the early apoptotic (Q2, 3.81%), late apoptotic (Q3, 3.42%), and necrotic (Q1, 2.57%) regions, reflecting the basal apoptotic status under normal culture conditions.

Conversely, treatment with Ag QDs at the IC₅₀ concentration for 48 h resulted in a pronounced shift in cell distribution (Fig. 5C). The proportion of viable cells markedly decreased to 60.4%, accompanied by a substantial rise in apoptotic populations. Evaluation of the flow cytometry data demonstrated a substantial increase in cell death, showing that 23.2% of the cells were undergoing early apoptosis, as identified by Annexin V positivity in the absence of propidium iodide (Q2). In parallel, delayed apoptotic events were observed in 14.4% of the population, characterized by simultaneous staining with both Annexin V and PI (Q3). Notably, only a limited increase in necrotic cells was observed (Q1, 8.63%), suggesting that membrane rupture and nonspecific cytotoxicity played a minor role.

Collectively, the significant accumulation of Annexin V‑positive cells following Ag QDs exposure demonstrates a strong induction of apoptosis in MCF-7 cells. The predominance of early and late apoptotic populations over necrotic cells indicates that Ag QDs predominantly induce programmed cell death rather than uncontrolled necrosis, thereby corroborating the apoptotic mechanism underlying their cytotoxic activity.

**Fig. 6:**
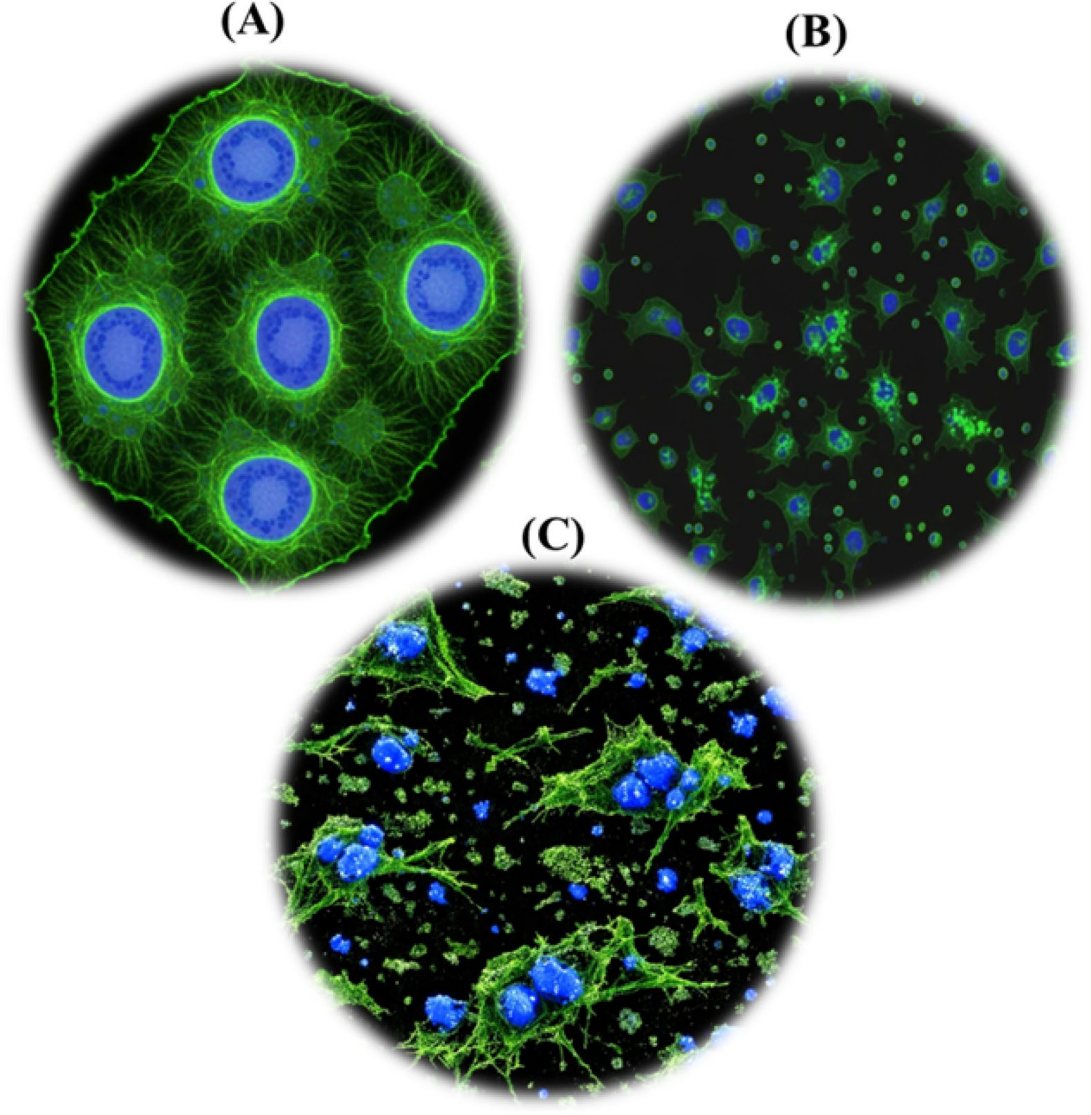
Confocal fluorescence microscopy of MCF‑7 cells. Nuclei (blue) and cytoskeleton (green) are shown. (**A**) Untreated control cells with intact nuclear morphology and organized cytoskeletal structure. (**B**) Cells treated with Ag QDs at the ½ IC₅₀ concentration displaying cytoskeletal disruption and nuclear condensation. (**C**) Positive control showing severe nuclear fragmentation and extensive cytoskeletal debris, indicative of advanced cell death.

Confocal fluorescence microscopy provided clear visual evidence of treatment‑dependent morphological and structural alterations in MCF‑7 cells (Fig. 6A-C). Control cells displayed a well‑preserved morphology, characterized by prominent, round nuclei with homogeneous staining and an extensive, highly organized cytoskeletal filament network. This structural integrity reflects normal nuclear organization and stable cytoskeletal architecture, indicative of healthy and viable cells (Fig. 6A).

In contrast, cells treated with Ag QDs at the ½ IC₅₀ concentration exhibited marked morphological alterations. Treated cells showed pronounced cell shrinkage, diminished cellular spreading, and disruption of intercellular contacts. These changes were accompanied by notable nuclear alterations, including chromatin condensation, nuclear size reduction, and irregular nuclear contours, all of which are hallmarks of apoptosis initiation. Simultaneously, the cytoskeletal structure underwent substantial reorganization, transitioning from an intact filamentous framework to a fragmented and disordered pattern, suggesting nanoparticle‑induced cytoskeletal destabilization (Fig. 6B).

The most extensive cellular damage was observed in the positive control group, where cells displayed severely condensed and fragmented nuclei along with a near‑complete collapse of the cytoskeletal network. The residual cytoskeletal signals appeared as scattered granular debris, consistent with advanced apoptotic progression or late‑stage cell death (Fig. 6C).

Overall, the confocal microscopy findings reveal a progressive decline in cellular integrity from untreated to Ag QD‑treated and positive control cells. These morphological changes provide clear visual evidence of the cytotoxic and apoptosis‑inducing effects of Ag QDs in MCF‑7 breast cancer cells, complementing the biochemical and flow cytometric evidence of programmed cell death.

### Effect of Ag QDs on mitochondrial membrane potential (ΔΨm)

The impact of green‑synthesized Ag QDs on mitochondrial membrane potential (ΔΨm) in MCF‑7 human breast cancer cells was investigated using the JC‑1 fluorescent probe, a sensitive indicator of mitochondrial polarization status. In healthy cells with intact mitochondria, JC‑1 accumulates in polarized mitochondria and forms red‑fluorescent J‑aggregates. Conversely, disruption of ΔΨm leads to dissociation of these aggregates into green‑fluorescent JC‑1 monomers.

**Fig. 7:**
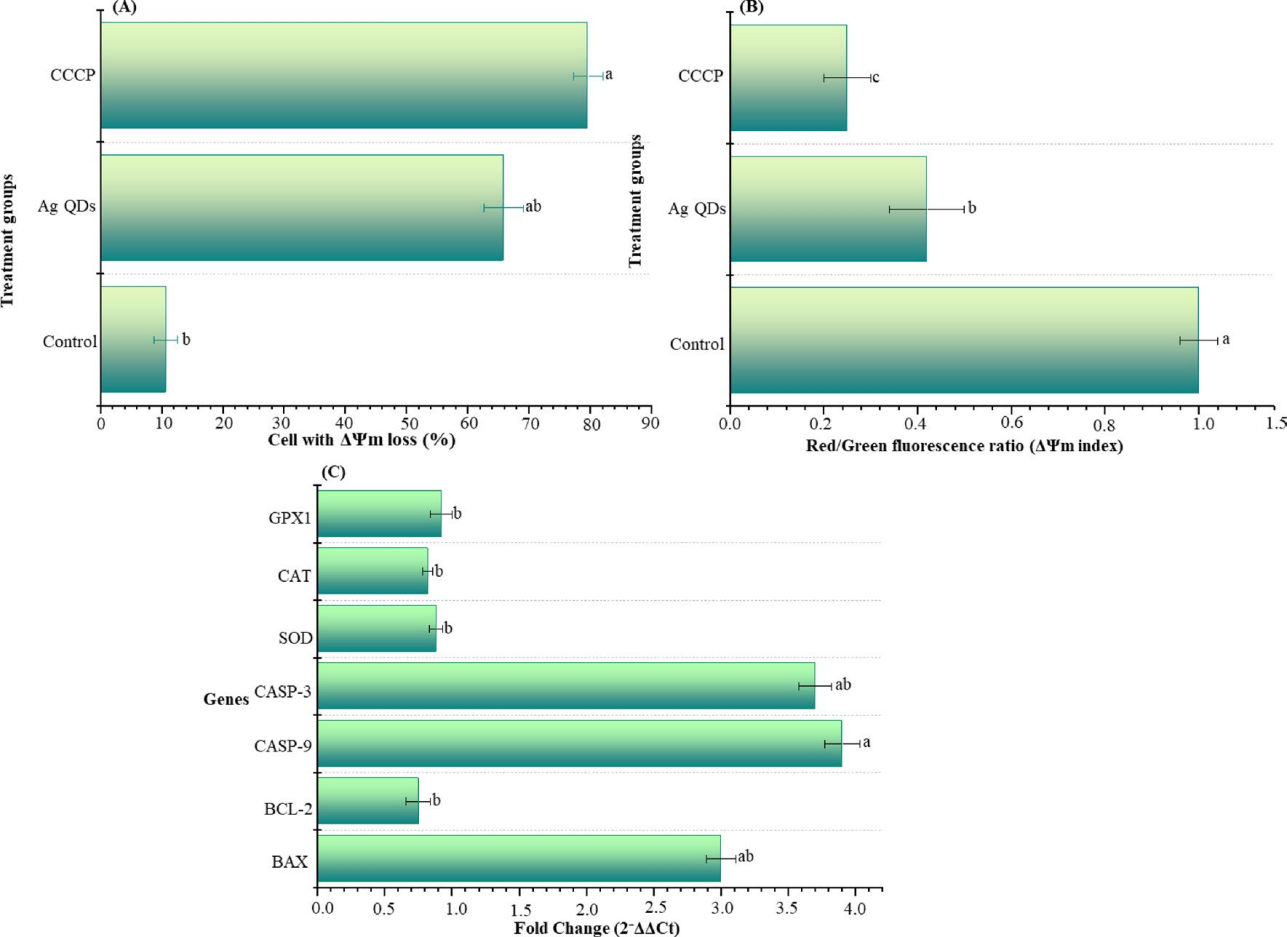
(**A**) Percentage of ΔΨm collapsed cells, (**B**) Red/Green fluorescence ratio (ΔΨm index) (*p < 0.001 vs. control), (**C**) Linking mitochondrial dysfunction to gene expression alterations in MCF-7 cells following Ag QDs exposure.

As illustrated in Fig. 7A, untreated control cells displayed strong red fluorescence accompanied by minimal green emission, reflecting preserved mitochondrial integrity and normal membrane potential. In contrast, MCF‑7 cells exposed to Ag QDs at the IC₅₀ concentration for 24 h exhibited a pronounced fluorescence shift from red to green, indicative of significant mitochondrial depolarization.

Quantitative analysis revealed that the JC‑1 red/green fluorescence intensity ratio markedly decreased from 1.00 ± 0.06 in control cells to 0.42 ± 0.05 following Ag QDs treatment (*p* < 0.001), consistent with marked loss of ΔΨm (Fig. 7B). As expected, treatment with the mitochondrial uncoupler carbonyl cyanide m‑chlorophenyl hydrazone (CCCP), used as a positive control, resulted in a further reduction of the red/green ratio to 0.25 ± 0.04, validating the sensitivity and reliability of the assay.

Flow‑cytometric quantification further supported these findings, showing that approximately 83.4 ± 3.8% of Ag QDs‑treated cells exhibited collapsed mitochondrial membrane potential, compared with only 9.7 ± 2.4% in untreated controls. Collectively, these findings suggest that Ag QDs markedly impair mitochondrial polarization in MCF‑7 cells.

Importantly, the observed ΔΨm loss was in line with the elevated intracellular ROS levels and the increased activities of caspase‑9 and caspase‑3 reported earlier, suggesting the involvement of a ROS‑associated mitochondrial apoptotic pathway.

Figure 7C further suggests a potential association between mitochondrial depolarization and the modulation of apoptosis‑ and redox‑related gene expression following Ag QDs exposure. The ΔΨm collapse was accompanied by changes in the expression of mitochondrial apoptotic regulators, characterized by upregulation of BAX and downregulation of BCL‑2, resulting in an increased BAX/BCL‑2 ratio, which is typically associated with mitochondrial outer membrane destabilization.

Taken together, the concurrent loss of ΔΨm and altered gene expression profiles indicate that Ag QDs may promote mitochondrial dysfunction and initiate intrinsic apoptotic signaling. However, additional protein‑level studies (e.g., cytochrome c release, Western blotting of caspase activation) will be required to definitively establish the mechanistic pathway.

## Discussion

The physicochemical analysis of the synthesized Ag QDs demonstrates their successful formation, colloidal stability, and uniform nanoscale architecture. A well‑defined surface plasmon resonance peak at 437 nm reflects the optical signature expected for quantum‑dot‑scale silver nanoparticles and indicates a highly dispersed colloidal system. The measured zeta potential (− 27.9 mV) further supports this stability, suggesting sufficient electrostatic repulsion to prevent particle aggregation under aqueous conditions. Crystallographic assessment reinforces these observations: the XRD patterns exhibit distinct diffraction peaks assigned to the (111), (200), (220), and (311) planes of face‑centered cubic silver, confirming both crystallinity and phase purity. The dominance of the (111) plane, together with the noticeable peak broadening, is characteristic of ultrafine crystallite formation typically associated with green‑synthesized silver nanostructures. In addition, the slight shifts observed in some diffraction peaks may arise from variations in the synthesis route or the presence of phytochemical compounds inherent to the plant extract, which can induce subtle lattice strain or surface interactions. Collectively, these results align well with previous reports on silver‑based nanostructures^9,10^.

A coherent size profile for the nanoparticles was also established. TEM imaging demonstrated well‑dispersed, nearly monodisperse spherical particles with an average core diameter of approximately 9.8 nm. Complementary DLS analysis revealed a hydrodynamic diameter of 8.10 nm, which aligns well with the expected size range for solvated quantum dots. Importantly, the slight variation observed between the particle sizes obtained from DLS (8.10 nm) and TEM (9.8 nm) is consistent with the fundamental differences in the measurement principles of these techniques. DLS reports the hydrodynamic diameter of nanoparticles dispersed in solution, which includes the solvated layer and reflects their dynamic behavior in the colloidal state. In contrast, TEM provides the physical, dry‑state diameter based on direct imaging of individual particles. Therefore, a minor discrepancy between these values is expected and does not indicate any inconsistency. Notably, both measurements fall within the same nanoscale range, confirming the overall uniformity and reliability of the particle size estimation. Together, these findings demonstrate that the synthesized Ag QDs possess desirable structural, optical, and colloidal features consistent with high‑quality quantum‑dot systems derived from green synthetic routes. The combined evidence from UV–Vis, DLS, zeta potential, XRD, and TEM analyses provides a comprehensive and mutually supportive characterization profile for the prepared Ag QDs. The exceptionally high phenolic (165 mg GAE/g DW) and flavonoid (125 mg Rutin/g DW) content of the *D. maritima* extract underscores the efficiency of microwave‑assisted extraction (MAE) in liberating bioactive compounds. MAE accelerates cell‑wall disruption and enhances solvent penetration, thereby maximizing the recovery of redox‑active metabolites^36,37^. This rich phytochemical profile provides a robust biochemical foundation for the observed antioxidant activities of both the crude extract and the green‑synthesized Ag QDs. Phenolics and flavonoids are well‑known for their ability to donate electrons or hydrogen atoms, chelate transition metals, and scavenge a wide spectrum of reactive oxygen and nitrogen species (ROS/RNS)^42,43^. The abundance of these compounds in the extract directly correlates with its strong performance in bulk redox assays such as FRAP and metal chelation.

A striking finding of this study is the superior radical‑scavenging activity of Ag QDs compared to the crude extract in ABTS (≈ 83% vs. 76%), DPPH (≈ 79% vs. 65%), and nitric oxide (≈ 57% vs. 46%) assays. This enhancement can be attributed to several nanoscale phenomena. First, the enormous surface‑area‑to‑volume ratio of quantum dots (typically < 10 nm) dramatically increases the number of accessible antioxidant sites per unit mass^] 35[^. Second, the metallic silver core can act as an electron reservoir, facilitating interfacial electron‑transfer reactions that quench radicals more efficiently than molecular antioxidants alone^30^. Third, the phytochemical capping layer not only stabilizes the nanoparticles but also presents a dense, oriented array of redox‑active functional groups (e.g., phenolic –OH, carbonyl) that can participate in concerted radical‑neutralization mechanisms. This synergistic interplay between the inorganic core and the organic shell is a hallmark of green‑synthesized metal‑based nanomaterials and explains why Ag QDs often outperform extracts in free‑radical assays^28^.

While Ag QDs excelled in radical‑scavenging tests, the crude extract exhibited stronger ferric‑reducing power (FRAP: 175 vs. 139 mmol Fe²⁺ equivalents) and metal‑chelating activity (65% vs. 46%). This divergence highlights the redistribution of phytochemicals during nanoparticle formation. During green synthesis, a substantial fraction of the extract’s redox‑active compounds is consumed in reducing Ag⁺ ions and subsequently anchored to the nanoparticle surface. These surface‑bound moieties are highly effective at interfacial electron transfer (hence the good radical‑scavenging results) but may be less accessible for bulk reduction of Fe³⁺ or chelation of free metal ions in solution. Conversely, the remaining soluble phytochemicals in the extract retain their native conformation and mobility, allowing them to engage more freely in homogeneous redox and chelation reactions^38^.

The moderate but significant NO‑scavenging capacity of Ag QDs (≈ 57%) is pharmacologically noteworthy. Nitric oxide is a key mediator of inflammation, and excessive NO production is implicated in chronic inflammatory disorders, neurodegenerative diseases, and diabetic complications^41^. The ability of Ag QDs to neutralize NO radicals, coupled with their high phenolic/flavonoid content, suggests a dual antioxidant‑anti‑inflammatory potential. Phenolic compounds are known to inhibit inducible nitric oxide synthase (iNOS) expression and directly scavenge NO radicals^42^. When these compounds are displayed on a nanoscale platform, their local concentration at biological interfaces may be amplified, potentially enhancing their anti‑inflammatory efficacy in vivo. This property positions green‑synthesized Ag QDs as promising candidates for nano‑therapeutic interventions aimed at oxidative‑stress‑associated inflammatory conditions.

The green‑synthesized Ag QDs, fabricated using *D. maritima* extract, exhibit potent cytotoxic and pro‑apoptotic activity against MCF‑7 breast cancer cells. The IC₅₀ of the Ag QDs (14.70 ± 0.68 µg/mL) closely approached that of cisplatin (12.4 ± 0.96 µg/mL), significantly surpassing the crude extract’s activity (62.5 ± 0.79 µg/mL). This significant enhancement emphasizes the pivotal role of nanostructuring in amplifying the biological efficacy of the constituent phytochemicals.

The pronounced cytotoxic effect is attributed to the QDs’ nanoscale dimensions and high surface‑to‑volume ratio, facilitating efficient cellular internalization via endocytic pathways (e.g., clathrin‑ or caveolae‑mediated endocytosis)^14,43^. Subsequent sustained interaction with organelles, especially mitochondria, leads to excessive ROS generation, mitochondrial membrane depolarization, and intrinsic apoptotic cascade activation, consistent with findings for other silver nanomaterials^44^. Conversely, the crude extract suffers from limited cellular uptake and rapid clearance. The conversion into a nanosized platform improves bioavailability and establishes synergistic interactions between the silver core and the surface phytochemicals, potentially enhancing redox activity.

This mechanistic hypothesis is supported by the ROS assay results, which showed that Ag QDs induced a 2.5‑fold increase in intracellular ROS, in contrast to the crude extract’s modest 1.2‑fold rise. This oxidative stress is consistent with the reported role of silver nanoparticles as pro‑oxidant agents that can promote the formation of superoxide anions (O₂˙⁻), hydrogen peroxide (H₂O₂), and hydroxyl radicals (˙OH) via surface‑mediated redox interactions^45^. The resulting elevated ROS levels may contribute to mitochondrial dysfunction and activation of downstream apoptotic signaling pathways. In line with this possibility, Ag QDs treatment resulted in a 3.7‑fold increase in caspase‑9 activity and a 3.6‑fold increase in caspase‑3 activity. Although these findings are consistent with involvement of a ROS‑associated intrinsic apoptotic response, definitive confirmation of mPTP opening or cytochrome c release would require protein‑level validation. The observed hierarchical pattern (caspase‑9 > caspase‑3) is compatible with a mitochondria‑linked apoptotic cascade and aligns with trends previously reported for silver nano‑systems. Notably, the caspase‑9 response closely approximated the positive control (4.0‑fold)^46^.

Flow cytometry using Annexin V/PI dual‑staining indicated that Ag QDs predominantly shift cells toward apoptotic rather than necrotic fates. Following treatment, the viable cell population decreased from 90.2% to 60.4%, accompanied by marked increases in early‑apoptotic (23.2%) and late‑apoptotic (14.4%) populations, while the necrotic fraction remained relatively low (8.63%).

Confocal microscopy further supported this apoptotic profile, revealing characteristic morphological features—such as cell shrinkage, chromatin condensation, nuclear fragmentation, and cytoskeletal disruption that are typically associated with apoptosis and consistent with downstream caspase-mediated structural alterations.

The robust cytotoxic and pro‑apoptotic effects observed for these Ag QDs are in agreement with recent studies on silver sulfide (Ag₂S) and other silver‑based quantum dots. For instance, Ag₂S QDs coated with 2‑mercaptopropionic acid have been reported as promising theranostic agents^47^, while NIR‑II emitting Ag‑based QDs have been shown to utilize ROS generation for dual imaging and therapeutic applications^48^. Our study advances this field by showing that the green synthesis route using *D. maritima* produces Ag QDs with notable cytotoxic potency, without the need for harsh chemical stabilizers. The phytochemical capping may contribute to both stabilization and potential bioactivity. While the observed cytotoxic response in MCF‑7 cells approached that of cisplatin under the tested conditions, broader validation across additional cancer and normal cell lines will be required. These findings nonetheless highlight the promise of these biosynthesized Ag QDs as candidates for future nano‑therapeutic development. Future work investigations should focus on in vivo validation, surface engineering for tumor targeting (e.g., folate conjugation), combination therapy studies, and a detailed mechanistic examination of downstream pathways (e.g., p53, Bax/Bcl‑2, MAPK, PI3K/Akt). Translational success will depend on thorough evaluation of long‑term biodistribution and off‑target effects, which requires further investigation alongside precise characterization of the phytochemical capping layer.

## Conclusion

This study presents a robust and environmentally benign green nanotechnology strategy for the synthesis of Ag QDs using the aqueous bulb extract of *D. maritima* (L.) Stearn, in which plant‑derived phytochemicals function simultaneously as reducing and stabilizing agents. This sustainable approach effectively circumvents the use of toxic chemical reductants and synthetic capping agents. Comprehensive physicochemical analyses verified the successful formation of well‑dispersed, colloidally stable Ag QDs with a narrow nanoscale size distribution (average hydrodynamic diameter ≈ 8.1 nm) and a moderately high negative surface charge (zeta potential ≈ − 27.9 mV), confirming efficient phytochemical capping and long‑term dispersion stability.

Comparative biological assessments revealed that both the crude *D. maritima* extract and the biosynthesized Ag QDs possess notable antioxidant properties, albeit with distinct functional specializations. While the crude extract exhibited superior electron‑donating capacity and metal‑chelating efficiency, as reflected by its higher FRAP value (≈ 175 mmol Fe²⁺ equivalents) and chelation activity (≈ 65%), the Ag QDs demonstrated markedly enhanced radical‑scavenging performance, achieving inhibition efficiencies of approximately 79% (DPPH), 83% (ABTS), and 57% (nitric oxide). This complementary antioxidant behavior underscores the role of nanoscale organization in redistributing and immobilizing phytochemicals at the nanoparticle surface, thereby selectively amplifying surface‑mediated redox interactions.

Beyond their antioxidant profile, *D. maritima*‑derived Ag QDs exhibited pronounced anticancer efficacy against MCF‑7 breast cancer cells, with an IC₅₀ value of 14.7 ± 0.68 µg mL⁻¹, closely approaching that of cisplatin (12.4 ± 0.96 µg mL⁻¹) and substantially lower than that of the crude extract (62.5 ± 0.79 µg mL⁻¹). Mechanistic investigations demonstrated that Ag QD exposure elicited a significant intracellular oxidative burst (≈ 2.5‑fold increase in ROS), which was associated with mitochondrial stress and activation of the intrinsic apoptotic pathway. This effect was supported by pronounced activation of caspase‑9 (≈ 3.7‑fold) and caspase‑3 (≈ 3.6‑fold), indicating a mitochondria‑dependent mode of programmed cell death.

Importantly, transcriptional profiling further supported these findings by revealing a coordinated modulation of apoptosis‑ and redox‑related genes. Treatment with Ag QDs resulted in the up‑regulation of pro‑apoptotic markers (BAX, CASP9, and CASP3), concomitant with the down‑regulation of the anti‑apoptotic gene BCL‑2, thereby markedly suggesting a shift in the BAX/BCL‑2 balance in favor of apoptotic commitment. In parallel, key antioxidant defense genes, including SOD1, CAT, and GPX1, were significantly suppressed, suggesting a potential reduction of the cellular redox buffering capacity and supporting the involvement of oxidative stress of apoptosis. These transcriptional alterations were fully consistent with the observed loss of mitochondrial membrane potential, Annexin V/PI flow cytometric profiles, and confocal microscopy findings, which collectively indicated apoptosis as the dominant mode of cell death with minimal necrotic contribution.

Taken together, this work advances current knowledge by showing that green synthesis using *D. maritima* yields multifunctional Ag QDs with cytotoxic potency approaching that of a clinically used chemotherapeutic agent, while maintaining a sustainable and chemically benign fabrication route. The combined presence of strong surface‑mediated antioxidant activity, elevated intracellular ROS generation, mitochondrial perturbation, and transcriptional modulation of apoptosis‑ and redox‑related genes suggests a coordinated cellular response to Ag QD exposure. Collectively, these findings highlight the potential of the synthesized Ag QDs as promising candidates for further investigation in nano‑therapeutic development.

To facilitate future clinical translation, subsequent studies should focus on in vivo efficacy and safety evaluation, detailed biodistribution and long‑term toxicity profiling, rational surface functionalization for tumor‑targeted delivery (e.g., folate or peptide conjugation), and exploration of combinational therapeutic strategies. Moreover, deeper investigation of downstream signaling pathways, including p53, MAPK, and PI3K/Akt networks, together with comprehensive molecular characterization of the phytochemical corona, may further clarify the mechanistic basis of their biological activity and support optimization of their translational potential.

## Data Availability

All data generated or analyzed during this study are included in this article. Further enquiries can be directed to the corresponding author.

## References

[1] Bozorgi, M., Amin, G., Shekarchi, M. & Rahimi, R. Traditional medical uses of *Drimia* species in terms of phytochemistry, pharmacology and toxicology. *J. Tradit Chin. Med.* **37**, 124–139. 10.1016/S0254-6272(17)30036-5 (2017). 29960283 10.1016/s0254-6272(17)30036-5

[2] Zhang, L. et al. Untargeted phenolic profiling and functional insights of the aerial parts and bulbs of *Drimia maritima* (L.) Stearn. *Plants* **11**, 600. 10.3390/plants11050600 (2022). 10.3390/plants11050600 PMC8912325 35270070

[3] Gvozdeva, Y. & Georgieva, P. Therapeutic potential of essential oils and their bioactive compounds against colon cancer: focus on colon-specific micro- and nanocarriers. *BioChem* **5**, 26. 10.3390/biochem5030026 (2025).

[4] Dehelean, C. A. et al. Plant-derived anticancer compounds as new perspectives in drug discovery and alternative therapy. *Molecules* **26**, 1109. 10.3390/molecules26041109 (2021). 33669817 10.3390/molecules26041109 PMC7922180

[5] Vallejo, M. J., Salazar, L. & Grijalva, M. Oxidative stress modulation and ROS-mediated toxicity in cancer: a review on in vitro models for plant-derived compounds. *Oxidative Med. Cell. Longev.* **2017** (4586068). 10.1155/2017/4586068 (2017). 10.1155/2017/4586068 PMC5674509 29204247

[6] Cancer accessed April 1, (n.d.). https://www.who.int/news-room/fact-sheets/detail/cancer (2025).

[7] . Global cancer burden growing, amidst mounting need for services, (n.d.). (accessed a April 1 2025); https://www.who.int/news/item/01-02-2024-global-cancer-burden-growing--amidst-mounting-need-for-services PMC11115397 38438207

[8] Zhou, J., Yang, Y. & Zhang, C. Y. Toward biocompatible semiconductor quantum dots: from biosynthesis and bioconjugation to biomedical application. *Chem. Rev.* **115**, 11669–11717. 10.1021/acs.chemrev.5b00049 (2015). 26446443 10.1021/acs.chemrev.5b00049

[9] Mariadoss, A. V. A. et al. Green synthesis, characterization and antibacterial activity of silver nanoparticles by Malus domestica and its cytotoxic effect on MCF–7 cell line. *Microb. Pathog*. 103609 10.1016/j.micpath.2019.103609. (2019). 31247255 10.1016/j.micpath.2019.103609

[10] Sathiyaseelan, A., Saravanakumar, K., Mariadoss, A. V. A. & Wang, M. H. Biocompatible fungal chitosan encapsulated phytogenic silver nanoparticles enhanced antidiabetic, antioxidant and antibacterial activity. *Int. J. Biol. Macromol.* **153**, 63–71. 10.1016/j.ijbiomac.2020.02.291 (2020). 32112842 10.1016/j.ijbiomac.2020.02.291

[11] Saravanakumar, K. et al. pH-sensitive release of fungal metabolites from chitosan nanoparticles for effective cytotoxicity in prostate cancer (PC3) cells. *Process. Biochem.*10.1016/j.procbio.2020.12.005 (2020/2021).

[12] Mariadoss, A. V. A., Saravanakumar, K., Sathiyaseelan, A. & Wang, M. H. Preparation, characterization and anti-cancer activity of graphene oxide–silver nanocomposite. *J. Photochem. Photobiol B: Biol.* **210**, 111984. 10.1016/j.jphotobiol.2020.111984 (2020). 10.1016/j.jphotobiol.2020.111984 32771914

[13] Saravanakumar, K., Mariadoss, A. V. A., Sathiyaseelan, A. & Wang, M. H. Synthesis and characterization of nano–chitosan capped gold nanoparticles with multifunctional bioactive properties. *Int. J. Biol. Macromol.* 16807. 10.1016/j.ijbiomac.2020.09.177 (2020). 10.1016/j.ijbiomac.2020.09.177 32980412

[14] An, X. et al. Oxidative cell death in cancer: mechanisms and therapeutic opportunities. *Cell Death Dis.* **15**, 556 (2024). 39090114 10.1038/s41419-024-06939-5 PMC11294602

[15] Niveria, K., Singh, P., Yadav, M. & Verma, A. K. Quantum dot-induced toxicity and biocompatibility. *Handb. II–VI Semicond. Sens. Radiat. Detect.* **1**, 181–211. 10.1007/978-3-031-19531-0_8 (2023).

[16] Lekic, N. et al. Differential oxidative stress responses to D-galactosamine–lipopolysaccharide hepatotoxicity based on real-time PCR analysis of selected oxidant/antioxidant and apoptotic gene expressions in rat. *Physiol. Res.* **60**, 549–558 (2011). 21401295 10.33549/physiolres.932041

[17] Shafiq, M. S. et al. Nanoparticle-induced oxidative stress in human cell lines: enzymatic biomarkers and gene expression disturbances. *Sch. Acad. J. Biosci.* **7**, 926–959 (2025).

[18] Szczyglewska, P., Feliczak-Guzik, A. & Nowak, I. Nanotechnology—general aspects: a chemical reduction approach to the synthesis of nanoparticles. *Molecules* **28**, 4932 (2023). 37446593 10.3390/molecules28134932 PMC10343226

[19] Sharma, N., Sharma, A. & Lee, H. J. The antioxidant properties of green carbon dots: a review. *Environ. Chem. Lett.* **23**, 1–49 (2025).

[20] Alvand, Z. M., Rajabi, H. R., Mirzaei, A., Masoumiasl, A. & Sadatfaraji, H. Rapid and green synthesis of cadmium telluride quantum dots with low toxicity based on a plant-mediated approach after microwave and ultrasonic assisted extraction: Synthesis, characterization, biological potentials and comparison study. *Mat. Sci. Eng. C*. **98**, 535–544. 10.1016/j.msec.2019.01.010 (2019). 10.1016/j.msec.2019.01.010 30813055

[21] Amir, M., Raheem, A., Jalil, S. U., Danish, M. & Ansari, M. I. Green-synthesized silver quantum dots mitigate arsenic translocation in spinach via glutathione-driven redox balance and modulation of stomatal-transpiration dynamics. *Plant. Stress*. **10**, 101138. 10.1016/j.stress.2025.101138 (2025).

[22] Eghlima, G., Aghamir, F., Mohammadi, M., Seyed Hajizadeh, H. & Kaya, O. Bioactive compounds and antimicrobial activities in Iranian *Crataegus persica* ecotypes for potential food and medicinal uses. *Food Sci. Nutr.* **13**, e4748. 10.1002/fsn3.4748 (2025). 39803229 10.1002/fsn3.4748 PMC11717551

[23] Rajabi, H. R., Alvand, Z. M. & Mirzaei, A. Sonochemical-assisted synthesis of copper oxide nanoparticles with the plant-mediated approach and comparative evaluation of biological activities. *Environ. Sci. Pollut Res.* **30**, 120236–120249. 10.1039/C9NJ03144H (2023). 10.1007/s11356-023-30684-5 37938488

[24] Silva, F. et al. A rapid and simplified DPPH assay for analysis of antioxidant interactions in binary combinations. *Microchem J.* **202**, 110801. 10.1016/j.microc.2024.110801 (2024).

[25] Bekdeser, B. & Apak, R. Colorimetric sensing of antioxidant capacity via auric acid reduction coupled to ABTS oxidation. *ACS Omega*. **9**, 11738–11746. 10.1021/acsomega.3c09134 (2024). 38497014 10.1021/acsomega.3c09134 PMC10938435

[26] Amin, K. M. Ferric reducing antioxidant parameter (FRAP) activity, separation of the flavonoids fraction and Folin–Ciocalteu assay of *Achillea oligocephala*. *Zanco J. Med. Sci.* **29**, 328–336. 10.15218/zjms.2025.034 (2025).

[27] Adeleke, A. A. et al. Synthesis and therapeutic potential of selected Schiff bases: in vitro antibacterial, antioxidant, antidiabetic, and computational studies. *ChemistrySelect* **9** (e202304967). 10.1002/slct.202304967 (2024).

[28] Moradi Alvand, Z., Rajabi, H. R., Mirzaei, A. & Masoumiasl, A. Ultrasonic- and microwave-assisted extraction as rapid and efficient techniques for plant-mediated synthesis of nanoparticles: green synthesis, characterization of zinc telluride and comparative biological evaluation. *New. J. Chem.* **43**, 15126–15138. 10.1039/C9NJ03144H (2019).

[29] Shafique, S. et al. Reimagining breast cancer treatment: MgO-IDA nanocomposites as a promising strategy for precision targeting of MCF-7 cells. *Results Chem.* **7**, 102660. 10.1016/j.rechem.2025.102660 (2025).

[30] Mohanta, Y. K. et al. Gold nanoparticle-mediated ROS generation and mitochondrial instability induced by *Ocimum* oil extracts against MCF-7 breast carcinoma. *RSC Adv.* **14**, 27816–27830. 10.1039/D4RA04807E (2024). 39224640 10.1039/d4ra04807e PMC11367626

[31] Asoudeh-Fard, A., Sis, F. F., Kamranjam, M., Soltanmohammadi, F. & Piri, H. Suppressive effects of *Tetraselmis suecica* algae extracts on MCF-7 breast cancer cell growth through apoptosis induction. *Iran. J. Sci. Technol.* **49**, 1–8. 10.1007/s40995-025-01893-z (2025).

[32] Gao, K. et al. TNF-α-induced mitochondrial dysfunction drives NLRP3/Caspase-1/GSDMD-mediated pyroptosis in MCF-7 cells. *Sci. Rep.* **14**, 25880. 10.18502/abi.v1i4.14719 (2024). 39468189 10.1038/s41598-024-76997-4 PMC11519391

[33] Abedi Tameh, F. et al. In vitro cytotoxicity of biosynthesized nanoceria using *Eucalyptus camaldulensis* leaf extract against MCF-7 breast cancer cells. *Sci. Rep.* **14**, 17465. 10.1038/s41598-024-68272-3 (2024). 39075175 10.1038/s41598-024-68272-3 PMC11286930

[34] Bustin, S. A. & Mueller, R. Real-time reverse transcription PCR (qRT-PCR) and its potential use in clinical diagnosis. *Clin. Sci.* **109**, 365–379 (2005). 10.1042/CS20050086 16171460

[35] Rovini, A. et al. Quantitative analysis of mitochondrial membrane potential heterogeneity in unsynchronized and synchronized cancer cells. *FASEB J.* **35**, e21148 (2020). 33196122 10.1096/fj.202001693R PMC7871195

[36] Tungmunnithum, D., Thongboonyou, A., Pholboon, A. & Yangsabai, A. Flavonoids and other phenolic compounds from medicinal plants for pharmaceutical and medical applications: an overview. *Medicines* **5**, 93. 10.3390/medicines5030093 (2018). 30149600 10.3390/medicines5030093 PMC6165118

[37] Spiridon, I., Bodirlau, R. & Teaca, C. A. Total phenolic content and antioxidant activity of plants used in traditional Romanian herbal medicine. *Cent. Eur. J. Biol.* **6**, 388–396. 10.2478/s11535-011-0028-6 (2011).

[38] Karimipour, M., Bagheri, M. & Molaei, M. One-pot rapid green photochemical synthesis of Ag₂S and Ag₂S–ZnS core–shell nanostructures. *J. Electron. Mater.* **48**, 2555–2562. 10.1007/s11664-019-06942-z (2019).

[39] Nordberg, J. & Arnér, E. S. J. Reactive oxygen species, antioxidants, and the mammalian thioredoxin system. *Free Radic Biol. Med.* **31**, 1287–1312. 10.1016/S0891-5849(01)00724-9 (2001). 11728801 10.1016/s0891-5849(01)00724-9

[40] McCord, J. M. The evolution of free radicals and oxidative stress. *Am. J. Med.* **108**, 652–659. 10.1016/S0002-9343(00)00412-5 (2000). 10856414 10.1016/s0002-9343(00)00412-5

[41] Kolak, U. et al. Phytochemical investigation of *Leontice leontopetalum* subsp. *ewersmannii* with antioxidant and anticholinesterase activities. *Rec Nat. Prod.* **5**, 309–313 (2011).

[42] Al-Dabbas, M. M. Antioxidant activity of different extracts from the aerial parts of *Moringa peregrina* from Jordan. *Pak J. Pharm. Sci.* **30**, 2151–2157 (2017). 29175784

[43] Nikazar, S. et al. Revisiting the cytotoxicity of quantum dots: an in-depth overview. *Biophys. Rev.* **12**, 703–718 (2020). 32140918 10.1007/s12551-020-00653-0 PMC7311601

[44] Al-Asiri, W. Y. et al. Cytotoxic and apoptotic effects of green synthesized silver nanoparticles via reactive oxygen species-mediated mitochondrial pathway in human breast cancer cells. *Cell. Biochem. Funct.* **42**, e4113 (2024). 39223765 10.1002/cbf.4113

[45] Mondal, J. et al. Promoting apoptosis in MCF-7 cells via ROS generation by quinolino-triazoles derived from one-pot telescopic synthesis. *ACS Med. Chem. Lett.* **15**, 1866–1874 (2024). 39563819 10.1021/acsmedchemlett.4c00289 PMC11571024

[46] Lee, Y. S. et al. Silver nanoparticles induce apoptosis and G2/M arrest via PKCζ-dependent signaling in A549 lung cells. *Arch. Toxicol.* **85**, 1529–1540 (2011). 21611810 10.1007/s00204-011-0714-1

[47] Vardar, D. O., Aydin, S., Hocaoglu, I., Acar, F. H. Y. & Basaran, N. Effects of silver sulfide quantum dots coated with 2-mercaptopropionic acid on genotoxic and apoptotic pathways in vitro. *Chem. -Biol Interact.* **291**, 212–219 (2018). 29958870 10.1016/j.cbi.2018.06.032

[48] Zhang, Z. et al. NIR-II silver-based quantum dots: synthesis and applications. *Nano Res.* **17**, 10620–10643 (2024).

